# Resveratrol Impairs Insulin Signaling in Hepatic Cells via Activation of PKC and PTP1B Pathways

**DOI:** 10.3390/ijms26157434

**Published:** 2025-08-01

**Authors:** Karla D. Hernández-González, Monica A. Vinchira-Lamprea, Judith Hernandez-Aranda, J. Alberto Olivares-Reyes

**Affiliations:** Laboratory of Signal Transduction, Department of Biochemistry, Center for Research and Advanced Studies of the National Polytechnic Institute, Cinvestav-IPN, Mexico City 07360, Mexico; daniela.hernandez@cinvestav.mx (K.D.H.-G.); monica.vinchira@unad.edu.co (M.A.V.-L.); juhernadez@cinvestav.mx (J.H.-A.)

**Keywords:** hepatic cells, resveratrol, insulin receptor, insulin resistance, protein kinase C, protein tyrosine phosphatase

## Abstract

Resveratrol (RSV), a polyphenol found in a variety of berries and wines, is known for its anti-inflammatory, anticancer, and antioxidant properties. It has been suggested that RSV may play a role in the regulation of metabolic disorders, including diabetes and insulin resistance. However, in recent years, it has been reported to completely inhibit Akt kinase function in liver cells. Akt is a central protein involved in the metabolic function of insulin and is regulated by the phosphatidylinositol-3-kinase (PI3K) pathway. In this study, we examined the effect of RSV on insulin-induced insulin receptor (IR) phosphorylation and proteins involved in the PI3K/Akt pathway in a hepatic cell model, clone 9 (C9), and in hepatoma cells, Hepa 1-6 (H1-6). In both cell lines, RSV inhibited tyrosine phosphorylation of IR and insulin-induced activation of Akt. We also evaluated the effect of RSV on the activation of protein tyrosine phosphatase 1B (PTP1B), which is associated with IR dephosphorylation, and found that RSV increased PTP1B-Tyr^152^ phosphorylation in a time- and concentration-dependent manner. Furthermore, we found that the protein kinase C (PKC) inhibitors BIM and Gö6976 prevented the inhibition of Akt phosphorylation by RSV and increased the phosphorylation of Ser/Thr residues in IR, suggesting that PKC is involved in the inhibition of the insulin pathway by RSV. Thus, classical PKC isoforms impair the PI3K/Akt pathway at the IR and GSK3 and GS downstream levels; however, IRS-Tyr^632^ phosphorylation remains unaffected. These results suggest that RSV can lead to insulin resistance by activating PTP1B and PKC, consequently affecting glucose homeostasis in hepatic cells.

## 1. Introduction

Resveratrol (RSV) (3,5,4′-trihydroxy-*trans*-stilbene), a polyphenol belonging to the stilbenoid group, is found in various red fruits, including grapes, blackberries, and certain nuts and berries [1,2,3]. This compound exhibits diverse beneficial properties, including anti-inflammatory, anticancer, antioxidant, and cardioprotective effects [1,3,4,5]. Of particular interest is its antioxidant activity, which can moderate or prevent high levels of reactive oxygen species (ROS) associated with metabolic syndrome (MetS), insulin resistance (InsR), type 2 diabetes (T2D), and cardiovascular diseases [6,7,8]. RSV is widely available in dietary supplements, including capsules, tablets, and pills, at doses ranging from 20 to 1400 mg per serving. It can be purchased in isolated form or in combination with other products [9]. The effects of RSV have been studied in various animal models and cell lines [10]; however, clinical studies are limited and have primarily focused on investigating its bioavailability, pharmacokinetics, and safety [11,12]. While some studies have reported beneficial effects, others have reported conflicting or adverse effects [3,13,14,15].

Although RSV has been associated with beneficial effects due to its antioxidant properties, its direct elimination activity is relatively low compared to other antioxidants, such as ascorbate, cysteine (Cys), and glutathione (GSH). Nevertheless, some reports have suggested that RSV may have pro-oxidant potential in some instances [16,17,18,19,20,21]. Previous studies have shown that the antioxidant effect of RSV in chronic treatments may be attributed to its ability to regulate the expression of antioxidant proteins, including catalase (CAT), glutathione peroxidase (GPX), and superoxide dismutase (SOD) [19,22,23]. Furthermore, it has been demonstrated that RSV can regulate ROS levels and improve metabolic conditions in diabetic models [10,24], as well as in situations involving MetS and InsR in animals [7,25,26]. It has also been reported to reduce blood glucose and plasma insulin levels, as well as improve insulin sensitivity and glucose uptake [27,28].

RSV has been reported to modulate metabolic functions in adipose tissue, skeletal muscle, and liver. In adipose tissue, RSV inhibits fat accumulation by downregulating the phosphoinositide 3-kinase (PI3K)/Akt pathway via estrogen receptor 1 (ESR1) [29]. Under insulin-resistant conditions, such as those induced by a high-fat/high-sugar diet (HFSD), RSV enhances the expression of insulin receptor substrate (IRS) and glucose transporter 4 (GLUT4) in visceral fat and reduces Akt phosphorylation at Ser^473^ [30]. In skeletal muscle, RSV restores the phosphorylation of insulin receptor (IR) and IRS and increases GLUT4 expression suppressed by tumor necrosis factor-α and lipopolysaccharide [31]. In skeletal C2C12 cells, RSV decreases Akt phosphorylation under insulin stimulation but enhances it in free fatty acid (FFA)-induced insulin resistance [32]. In the liver of high-fat diet-fed mice, chronic RSV treatment increases the phosphorylation of Akt and glycogen synthase kinase-3β (GSK3β) and upregulates IRS expression without altering its phosphorylation state [33,34]. RSV also inhibits the activation of PI3K and Akt in various liver cells and primary hepatocytes [35,36]. These findings highlight the potential of RSV to improve insulin sensitivity and metabolic functions. However, as reported, the effects on normal InsR-deficient cells in metabolic tissues are consistent with the inhibition of PI3K/Akt, a central component of the insulin signaling pathway involved in glucose uptake, glycogen synthesis, and lipid metabolism [37,38]. The implications of inhibiting PI3K/Akt under normal or non-InsR conditions remain unclear and warrant further investigation to understand its impact on metabolism.

PI3K is activated upon insulin binding to IR, leading to autophosphorylation and phosphorylation of its substrates, including IRS [37,39,40]. IRS functions as a scaffolding protein that interacts with the p85 regulatory subunit of PI3K, resulting in a conformational change that activates the enzyme [38,41,42]. PI3K then catalyzes the phosphorylation of phosphatidylinositol 4,5–bisphosphate (PIP_2_) to generate phosphatidylinositol 3,4,5–triphosphate (PIP_3_), which serves as a scaffolding site for the recruitment of Akt to the plasma membrane. Akt is subsequently activated via phosphorylation at Thr^308^ and Ser^473^ by PDK1 and mTORC2 (PDK2), respectively [43]. Akt triggers several metabolic processes, including glycogen synthesis, protein synthesis, and adipogenesis [37]. Insulin is critical for the regulation of glucose homeostasis by inhibiting glucose production and promoting glucose storage, particularly in the liver [37]. Glycogen synthesis is crucial for maintaining glucose homeostasis, as it regulates glucose excess; alterations in hepatic IR and signaling result in elevated glucose levels and hyperinsulinemia [44,45,46].

To evaluate the role of RSV under normal and pathological conditions, the present study used two types of hepatic cell lines: Clone 9 (C9) and Hepa1-6 (H1-6). C9 cells are a normal rat hepatic cell model that retains an epithelial phenotype [47] and exhibits the endogenous expression of IRs [41,48,49]. Furthermore, C9 cells provide a valuable model for studying the molecular mechanisms underlying insulin signaling in hepatic cells [41,50,51,52]. H1-6 cells are a mouse cell line derived from hepatoma, which serves as a valuable model for understanding tumor biology, testing potential therapies, and investigating drug responses in hepatocellular carcinoma, including the action of RSV [53,54]. Although H1-6 cells respond to insulin by increasing the activation of IR and Akt (pSer^473^), recent studies suggest that they exhibit abnormal post-IR signaling, characterized by reduced and insulin-insensitive glucose production [55].

Thus, the present study aimed to evaluate the effect of RSV on the phosphorylation of key proteins involved in insulin signaling, including IR, IRS-1, Akt, glycogen synthase kinase-3α (GSK3α), and GSK3β, and glycogen synthase (GS). Our findings suggest that RSV impairs insulin-stimulated IR phosphorylation, which decreases the activation of Akt and its associated downstream proteins involved in glycogen synthesis. This effect may be attributed to the activation of protein tyrosine phosphatase 1B (PTP1B), as evidenced by the increased phosphorylation induced by RSV. Furthermore, inhibition of protein kinase C (PKC) restored insulin-stimulated phosphorylation of Akt and extracellular signal-regulated kinase 1/2 (ERK1/2), suggesting that RSV may induce InsR through PKC activation.

## 2. Results

### 2.1. Insulin Signaling Pathway in Liver Cells

**Figure 1:**
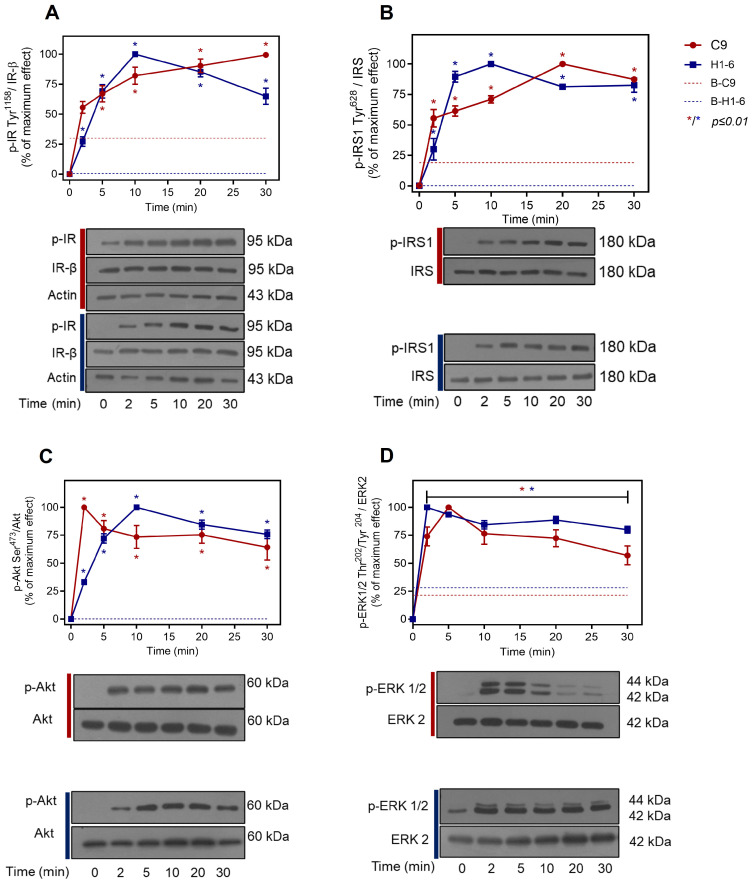
Activation of the insulin pathway in hepatic cells. C9 and Hepa 1-6 cells were stimulated with 100 nM insulin for the indicated times. Total cell lysates were separated by SDS-PAGE and analyzed by immunoblotting using anti-p-IR-Tyr^1158^ (**A**), anti-p-IRS-1-Tyr^628^ (**B**), anti-p-Akt-Ser^473^ (**C**), and anti-p-ERK1/2-Thr^202^/Tyr^204^ (**D**), as described in Materials and Methods. Data represent the mean ± S.E.M. of five independent experiments, and the panels below show representative blots. Western blots were also probed for total IR, IRS, Akt, ERK, and actin, all showing equal loading. Vertical lines represent the S.E.M. values. * p-value ≤ 0.01 vs. basal phosphorylation (**A**–**D**). C9, clone 9 cells (red circles); H1-6, Hepa 1-6 cells (blue squares); B-C9, basal phosphorylation of C9 (red dotted line); B-H1-6, basal phosphorylation of H1-6 (blue dotted line).

First, we assessed the effects of insulin stimulation on clone 9 (C9) and Hepa 1-6 (H1-6) cells. To determine the activation status of the PI3K/Akt signaling pathway, we analyzed the specific phosphorylation of key proteins in the IR/IRS/Akt pathway. Upon stimulation of C9 cells with 100 nM insulin for 0–30 min, we observed a significant increase in IR phosphorylation at Tyr^1158^ after 5 min (~2.5–fold increase over the control), which reached a peak at 20–30 min (Figure 1A, red line). Similarly, insulin triggered a rapid and sustained increase in IRS-1 Tyr^628^ phosphorylation (corresponding to the rat sequence). This increase began after 2 min (~2–fold increase over the control), reached a maximum at 20 min, and persisted for at least 30 min (Figure 1B, red line). Consistently, Akt was activated by insulin, reaching a maximum level of phosphorylation at 2 min, and persisted for the next 30 min (Figure 1C, red line). Interestingly, a similar response was observed in H1-6 cells when stimulated with 100 nM insulin for 0 to 30 min, showing consistent IR, IRS, and Akt phosphorylation levels (Figure 1A–C, blue line). In all cases, a rapid response was observed, starting within 2 min (~25–fold increase over the control), peaking at 5 min for IRS-1 Tyr^628^, and 10 min for IR Tyr^1158^ and Akt Ser^473^ (Figure 1A–C, blue line). Thereafter, a slight decline was observed for up to 60 min (Supplementary Figure S1A–C).

The activation of the MAPK/ERK1/2 pathway, which regulates the proliferative actions of insulin, was also examined in both hepatic cell lines. In C9 cells, the phosphorylation of ERK1/2 at Thr^202^/Tyr^204^ showed a significant increase at 2 min (~3–fold increase compared to the control), reaching maximal activation at 5 min (Figure 1D, red line). Conversely, in H1-6 cells, insulin-induced ERK1/2 Thr^202^/Tyr^204^ phosphorylation was relatively sustained, reaching a maximum at 2 min (~4–fold increase) compared with the control (Figure 1D, blue line). Both cell lines exhibited sustained ERK1/2 phosphorylation for up to 30 min. Notably, differences in the baseline phosphorylation of the evaluated proteins were evident between the two cell lines. Specifically, C9 cells displayed higher baseline phosphorylation of IR and IRS than H1-6 cells, whereas H1-6 cells exhibited higher ERK1/2 phosphorylation levels. However, the baseline phosphorylation of Akt Ser^473^ was comparable between the two cell types. Regarding the insulin response, C9 cells displayed the more rapid activation of Akt, whereas H1-6 cells displayed a more rapid response in the phosphorylation of ERK1/2.

Despite the observed differences, the changes in the phosphorylation of IR, IRS, Akt, and ERK1/2 following insulin stimulation indicated that both hepatic cell lines responded as expected to the hormone, activating the two primary signaling pathways, PI3K/Akt and MAPKs. Consequently, both hepatic cell models are suitable for studying the effects of RSV on insulin signaling.

### 2.2. Resveratrol Desensitizes Insulin Signaling

**Figure 2:**
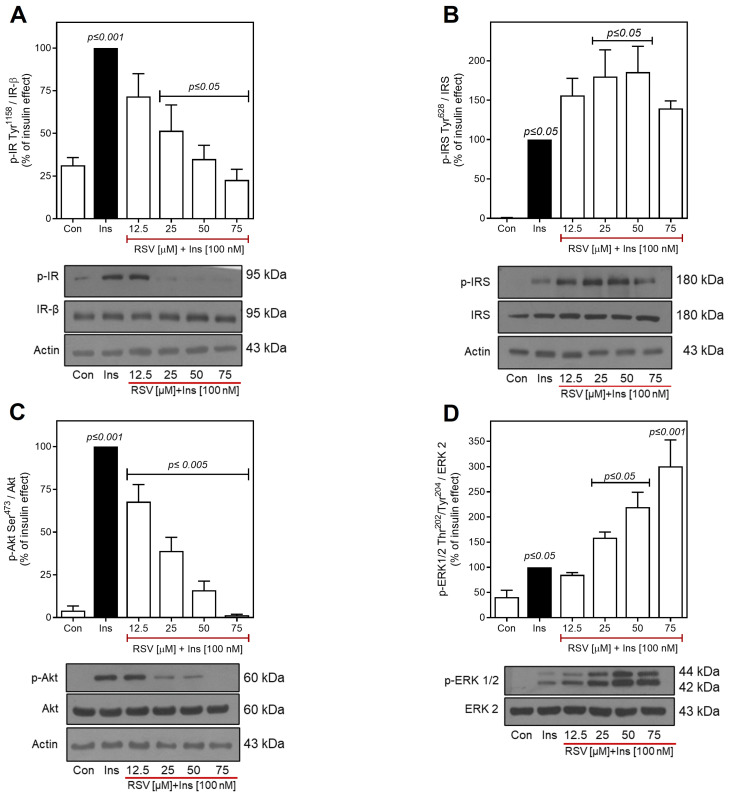
Effect of RSV on insulin-induced phosphorylation of IR/IRS/Akt and ERK in C9 cells. C9 cells were pretreated with different concentrations of RSV (12.5–75 µM as indicated) and stimulated with 100 nM insulin for an additional 10 min. Under all experimental conditions, untreated cells were used as controls. Total cell lysates were separated by SD-PAGE and analyzed by immunoblotting with anti-p-IR-Tyr^1158^ (**A**), anti-p-IRS-1-Tyr^628^ (**B**), anti-p-Akt-Ser^473^ (**C**), or anti-p-ERK1/2-Thr^202^/Tyr^204^ (**D**), as described in Materials and Methods. Data represent the mean ± S.E.M. of five independent experiments, and the panels below show representative immunoblots. Western blots were also probed for total IR, IRS, Akt, and ERK1/2, which showed equal loading. Vertical lines represent the S.E.M. The *p*-value indicates: Ins vs. control (**A**–**D**); RSV [12.5–75 µM] + Ins [100 nM] vs. Ins (**A**–**D**).

After confirming the insulin response in both cell types, assays were performed to investigate the effects of RSV on insulin signaling. Previous reports have indicated that RSV inhibits the insulin-induced phosphorylation of Akt at Ser^473^ in different hepatic cell lines and primary hepatocytes [35,36]. Therefore, we investigated whether RSV impairs insulin-induced Akt activation in C9 and H1-6 cells. Cells were pretreated for 30 min with increasing concentrations of RSV (ranging from 12.5 to 75 μM) and subsequently stimulated with 100 nM of insulin for 10 min. Our results in C9 cells revealed that RSV inhibited the phosphorylation of IR Tyr^1158^ and Akt Ser^473^ in a concentration-dependent manner (Figure 2A and Figure 2C, respectively). As shown in Figure 2A, the phosphorylation of IR at Tyr^1158^ decreased significantly with 25 μM of RSV (~25% of the insulin effect) and was completely inhibited by 50–75 μM of RSV treatment. In contrast, Akt Ser^473^ phosphorylation decreased significantly with 12.5 μM of RSV (~30% of the insulin effect, Figure 2C). Despite the observed differences, increased RSV concentration similarly impaired IR Tyr^1158^ and Akt Ser^473^ phosphorylation, with IC_50_ values of 27.5 μM and 19.1 μM, respectively (Supplementary Figure S2A). As RSV inhibited insulin-stimulated phosphorylation of IR/Akt, it was expected that RSV would have a similar effect on IRS-1 Tyr^628^ phosphorylation. Surprisingly, the effect of RSV was contrary to what was expected. RSV induced a significant concentration-dependent increase in IRS-1 Tyr^628^ phosphorylation (~2–fold increase over the insulin effect), with the maximum effect observed between 25 and 50 µM of RSV (Figure 2B). Similarly, RSV increased insulin-stimulated phosphorylation of ERK1/2 Thr^202^/Tyr^204^. This increase was significant at 25 μM (~1.8–fold increase over the insulin effect), and the maximum effect was observed at 50–75 μM of RSV (~3–fold increase over the insulin effect) (Figure 2D).

**Figure 3:**
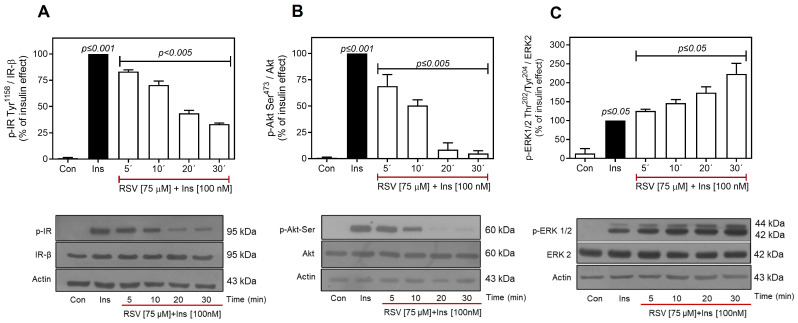
RSV affects time-dependent insulin-induced phosphorylation of IR/Akt. C9 cells were pretreated with RSV (75 µM) for the indicated times and then stimulated with 100 nM insulin for an additional 10 min. Under all experimental conditions, untreated cells were used as controls. Total cell lysates were separated by SD-PAGE and analyzed by immunoblotting with anti-p-IR-Tyr^1158^ (**A**), anti-p-Akt-Ser^473^ (**B**), or anti-p-ERK1/2-Thr^202^/Tyr^204^ (**C**), as described in Materials and Methods. Western blots were also probed for total IR, Akt, and ERK1/2 showing equal loading. Data represent the mean ± S.E.M. of five to six individual experiments, and the panels below show representative immunoblots. Vertical lines represent the S.E.M. The *p*-value indicates Ins vs. control (**A**–**C**); RSV (5–30 min) + Ins [100 nM] vs. Ins (**A**–**C**).

Subsequently, the effect of RSV on IR phosphorylation was analyzed over time by pre-incubating the cells with 75 μM of RSV for 5–30 min, followed by the addition of 100 nM insulin for 10 min. As shown in Figure 3A, insulin-stimulated IR Tyr^1158^ phosphorylation decreased significantly after 5 min of incubation (~25%), reaching a maximum reduction of 75% after 30 min of RSV treatment. Furthermore, insulin-induced phosphorylation at Ser^473^ decreased at 5 min (~30% of the insulin effect) and was completely inhibited after 20 min of RSV pretreatment (Figure 3B). Although there were temporal differences in the significant decreases in IR Tyr^1158^ and Akt Ser^473^ phosphorylation caused by RSV, they exhibited similar IC_50_ values (IC_50_ of ~27.5 µM, p-IR; IC_50_ of ~19.1 µM, p-Akt). Consistent with our results, RSV has been reported to inhibit insulin-activated PI3K (IC_50_ = 25 µM) [36], suggesting that inhibition of Akt phosphorylation by RSV depends on its upstream effects at the IR level. Next, we evaluated the effect of RSV on insulin-induced ERK1/2 phosphorylation. As shown in Figure 3C, RSV, as expected, increased ERK1/2 phosphorylation, which was initiated at 5 min and reached maximal activation (~2.5–fold increase over the insulin effect) after 30 min of RSV prestimulation.

**Figure 4:**
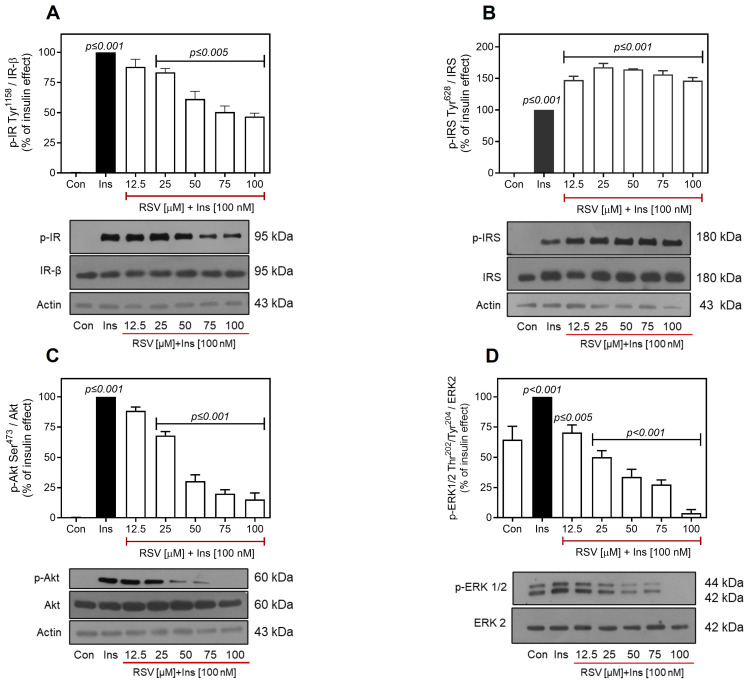
Effect of RSV on insulin-induced phosphorylation of IR/IRS/Akt and ERK in Hepa 1-6 cells. Hepa 1-6 cells were pretreated with different concentrations of RSV (12.5–100 µM as indicated) and then stimulated with 100 nM insulin for an additional 10 min. Under all experimental conditions, untreated cells were used as controls. Total cell lysates were separated by SD-PAGE and analyzed by immunoblotting with anti-p-IR-Tyr^1158^ (**A**), anti-p-IRS-1-Tyr^628^ (**B**), anti-p-Akt-Ser^473^ (**C**), or anti-p-ERK1/2-Thr^202^/Tyr^204^ (**D**), as described in Materials and Methods. Western blots were also probed for total IR, IRS, Akt, and ERK1/2 showing equal loading. Data represent the mean ± S.E.M. of five to six individual experiments, and the panels below show representative immunoblots. Vertical lines represent the S.E.M. The *p*-value indicates Ins vs. control (**A**–**D**); RSV [12.5–100 µM] + Ins [100 nM] vs. Ins (**A**–**D**).

A similar effect of RSV on insulin-induced IR Tyr^1158^/Akt Ser^473^ phosphorylation was observed in H1-6 cells. At 25 µM RSV, there was a decrease of ~15% in IR and ~25% in Akt phosphorylation. However, when the concentration of RSV was increased to 75–100 µM, a maximal decrease of ~50% in IR and ~80% in Akt was observed (Figure 4A and Figure 4C, respectively). Nevertheless, the IC_50_ values for the effect of RSV on IR and Akt phosphorylation indicated a reduced sensitivity to RSV in H1-6 cells compared to C9 cells (IC_50_ = 70.9 µM for IR and 35.6 µM for Akt vs. IC_50_ = 27.5 µM for IR and 19.1 µM, respectively, as shown in Supplementary Figure S2A,B). The observed differences in the effects of RSV on IR and Akt suggest that an alternative mechanism may negatively regulate the phosphorylation of IR and Akt in C9 cells, potentially accounting for the increased susceptibility to RSV. RSV may mediate the dephosphorylation of IR and Akt by activating protein phosphatases, such as PTP1B and protein phosphatase 2A (PP2A), which negatively regulate the activity of Akt and IR, respectively [56,57,58,59].

In H1-6 cells, RSV induced an increase in IRS-1 Tyr^628^ phosphorylation levels (~1.8–fold increase over the insulin effect) (Figure 4B), which was similar to the increase observed in C9 cells (Figure 2B). However, in these cells, RSV produced a different effect on ERK1/2 phosphorylation compared to that in C9 cells. While RSV increased phosphorylation in C9 cells, it significantly decreased ERK1/2 Thr^202^/Tyr^204^ phosphorylation in H1-6 cells in a concentration-dependent manner, with an IC_50_ value of 26.7 μM (Figure 4D; Supplementary Figure S2B). These results are consistent with previous reports demonstrating that RSV inhibits the ERK1/2 pathway in cancer cells, thereby contributing to its anticancer effects [5,58].

Although puzzling, similar observations regarding the effect of RSV on ERK activation [60] or inhibition [35,58] have been reported in different cellular contexts [61,62]. Inhibition of ERK1/2 mediated by RSV has been demonstrated in multiple cancer cell lines, including rat hepatoma cells (H4IIE), through mechanisms involving activation of PP2A and PTEN [58,61,63], as well as dissociation of IRS, PI3K, and Grb2 [35,58,64]. In contrast, RSV has been shown to activate ERK1/2 in prostate, breast [65], and ovarian cancer cells [66], among other cancers [67]. Constitutive activation of the MAPK pathway is essential for maintaining the malignant phenotype of cancer cells; however, short-term activation leads to apoptosis [62,68], which may explain the pro-apoptotic effect of RSV through ERK1/2 activation. Other studies have reported concentration-dependent activation or inhibition of ERK by RSV; at low concentrations (<10 µM), RSV activates ERK1/2, whereas at concentrations in the range of 50–100 µM, it inhibits it [69].

The differences in the effects of RSV on ERK1/2 phosphorylation between the two cell lines may result from metabolic differences in the cells and changes in the expression and functionality of proteins, including PP2A and PTEN, particularly in cancer cells such as H1-6 [58,70,71].

### 2.3. Resveratrol Negatively Regulates the Insulin Pathway Downstream of Akt

**Figure 5:**
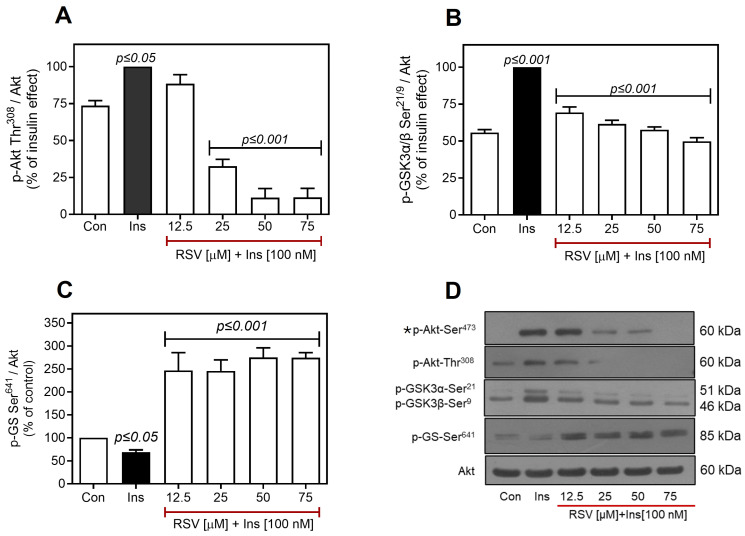
RSV negatively regulates the insulin pathway downstream of Akt. C9 cells were pretreated with different concentrations of RSV (12.5–75 µM as indicated) and then stimulated with 100 nM insulin for an additional 10 min. Under all experimental conditions, untreated cells were used as controls. Total cell lysates were separated by SD-PAGE and analyzed by immunoblotting with anti-p-Akt-Thr^308^ (**A**) or anti-p-GSK3α/β-Ser^21/9^ (**B**) and anti-p-GS-Ser^641^ (**C**), as described in Materials and Methods. Representative blots of Akt, GSK3, and GS phosphorylation are shown (**D**). * The same blot was used in Figure 2C, as it corresponds to the same experiment. Western blots were also probed for total Akt showing equal loading. Data represent the mean ± S.E.M. of five to six individual experiments, and the panels below show representative immunoblots. Vertical lines represent the S.E.M. The *p*-value indicates Ins vs. control (**A**–**D**); RSV [12.5–75 µM] + Ins [100 nM] vs. Ins (**A**–**D**).

Although the effects of RSV on IR Tyr^1158^ and Akt Ser^473^ phosphorylation in C9 cells were similar, the inhibition of Akt Ser^473^ phosphorylation was not entirely dependent on IR inhibition, particularly in H1-6 cells. These observations suggest that RSV directly regulates other intermediate proteins in the insulin pathway. Therefore, we assessed the effect of RSV downstream of Akt. Given previous reports indicating that RSV can directly inhibit PI3K [36,64,72] and that other bioactive compounds structurally related to RSV can inhibit Akt phosphorylation at Thr^308^ [73], we initially evaluated the effect of RSV on Akt Thr^308^ phosphorylation in C9 cells. As shown in Figure 5A,D, incubation of cells with increasing concentrations of RSV, followed by the addition of 100 nM of insulin for 10 min, resulted in a decrease in insulin-induced phosphorylation of Akt at Thr^308^. This effect was significant at 25 µM and reached maximal inhibition between 50 and 75 µM of RSV pretreatment, with an IC_50_ of 21.6 µM (Supplementary Figure S2A).

Subsequently, we evaluated the effects of RSV on GSK3α/β and GS, which are critical proteins involved in insulin-mediated regulation of hepatic glycogen synthesis. GSK3α/β is a Ser/Thr kinase that inhibits glycogen synthesis through GS phosphorylation and is negatively regulated by Akt. As expected, RSV prevented insulin-induced GSK3α/β phosphorylation (Figure 5B,D), particularly that of GSK3α (Supplementary Figure S3A,B). The decrease in GSK3α-Ser^21^ phosphorylation suggests that RSV maintains GSK3α active, which then phosphorylates GS at Ser^641^ (~3–fold increase compared to the effect of insulin, Figure 5C,D) and inactivates it, thereby reducing glycogen synthesis [74,75]. These findings indicate that RSV may modulate subsequent metabolic processes, such as glycogen synthesis, by inhibiting IR activity in hepatic C9 cells.

### 2.4. Resveratrol Downregulates the IR/Akt Pathway via PKC

Following the evaluation of the RSV effect on insulin metabolic functions and the confirmation of pathway inhibition at the level of IR and downstream glycogen synthesis, our investigation focused on uncovering the mechanisms by which RSV inhibits IR activity. It is well established that among the regulatory mechanisms of the insulin pathway at the level of IR/IRS is the increased expression and activity of Ser/Thr protein kinases and protein tyrosine phosphatases (PTPs), which specifically dephosphorylate IR/IRS. The phosphorylation of Ser/Thr residues on IR/IRS results in diminished Tyr phosphorylation and the catalytic activity of IR. This process also leads to the dissociation of IR from IRS and downstream proteins, resulting in reduced Akt activity [76,77,78,79].

**Figure 6:**
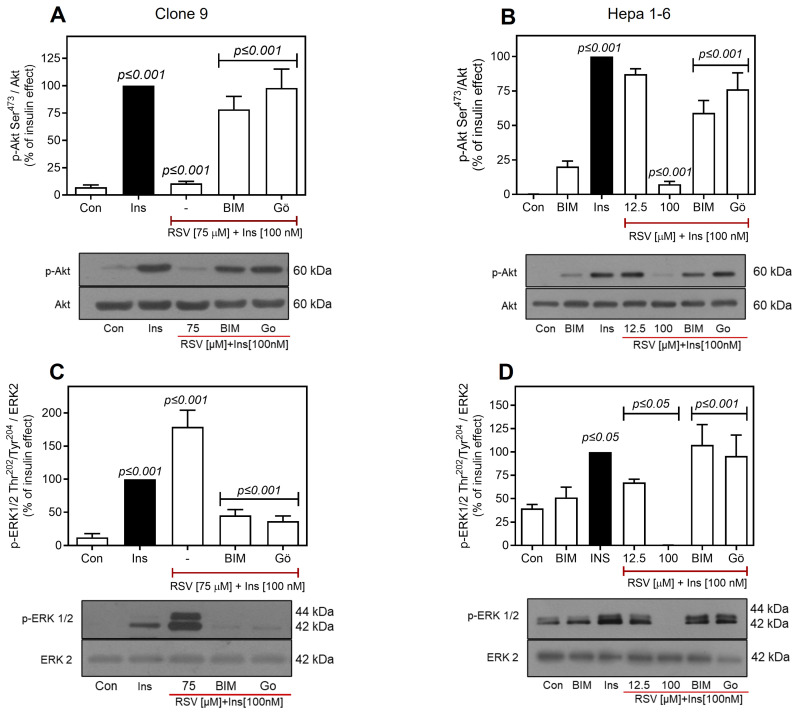
Role of PKC in RSV-mediated regulation of insulin signaling. C9 and Hepa 1-6 cells were pretreated with or without 1 µM BIM or 100 nM Gö6976 for 30 min, and then treated with different concentrations of RSV (as indicated) for 30 min. Finally, the cells were stimulated with 100 nM insulin for an additional 10 min. Under all experimental conditions, untreated cells were used as controls. Total cell lysates were separated by SD-PAGE and analyzed by immunoblotting with anti-p-Akt-Ser^473^ (**A**,**B**) or anti-p-ERK1/2-Thr^202^/Tyr^204^ (**C**,**D**), as described in Materials and Methods. Western blots were also probed for total Akt and ERK2, which showed equal loading. Data represent the mean ± S.E.M. of five to six individual experiments, and the panels below show representative immunoblots. Vertical lines represent the S.E.M. The *p*-value indicates Ins vs. control (**A**–**D**); RSV [75 µM] + Ins [100 nM] (**A**,**C**) or RSV [12.5 and 100 µM] + Ins [100 nM] vs. Ins (**B**,**D**); BIM or Gö vs. RSV [75 µM] + Ins [100 nM] (**A**,**C**) or RSV [100 µM] + Ins [100 nM] (**B**,**D**).

PKC isoforms have been shown to negatively regulate IR/IRS activity by phosphorylating multiple Ser/Thr residues near IR autophosphorylation sites and along the IRS structure [76,80,81,82]. Studies have shown that increased PKC activity or expression is associated with InsR and T2D [79,83,84]. Consequently, this study investigated the potential role of PKC in RSV-mediated negative regulation of the insulin pathway. To assess the role of PKC in the inhibition of insulin-mediated IR/Akt phosphorylation by RSV, the effects of two PKC inhibitors were examined: bisindolylmaleimide I (BIM), a highly selective, cell-permeable, and reversible inhibitor of classical and novel PKC isoforms [77], and Gö6976, a selective inhibitor of PKCα and PKCβ isoforms [85,86]. As shown in Figure 6A, pretreatment of C9 cells with 1 μM BIM or 100 nM Gö6976 for 30 min prevented the effect of 75 µM RSV on insulin-induced Akt Ser^473^ phosphorylation to the same extent. Furthermore, BIM inhibited the action of RSV in a concentration-dependent manner, although this inhibition was less pronounced at the highest concentration of RSV (75 μM) (Supplementary Figure S4A). Moreover, the inhibition of PKC with BIM in C9 cells restored GSK3-Ser^21/9^ phosphorylation (Supplementary Figure S4B), which was previously observed to be inhibited by RSV (Figure 5B).

Consistent with the data obtained in C9 cells, both inhibitors partially prevented the effects of RSV in H1-6 cells. Gö6976 inhibited the effect of RSV on insulin-induced Akt phosphorylation by ~76.2 ± 11.8%, whereas BIM significantly reduced it by ~59.17 ± 8.8% (Figure 6B). These findings suggest that the inhibitory effect of RSV may be attributed to classical PKC isoforms. This conclusion was further supported by the observation that the Gö6976 inhibitor largely restored insulin-induced phosphorylation and prevented RSV-induced Akt inhibition in both cell types.

The effect of PKC inhibition on insulin-mediated ERK1/2 phosphorylation regulated by RSV was also determined. As shown in Figure 6C, both PKC inhibitors exhibited similar effects on C9 cells, preventing RSV-induced increase in ERK1/2 phosphorylation and even promoting a decrease (~50–60%) below that of insulin alone. The decrease in phosphorylated ERK levels between the inhibitor treatments was not statistically significant, suggesting the potential involvement of classical PKC isoforms in RSV-induced ERK1/2 inhibition. Additionally, increasing concentrations of BIM (ranging from 12.5 to 75 µM) reduced ERK1/2 phosphorylation induced by insulin and RSV, as expected (Supplementary Figure S4C). Similarly, in H1-6 cells, both PKC inhibitors completely prevented the inhibitory effect of 100 µM RSV on insulin-induced ERK1/2 phosphorylation (Figure 6D). No significant differences were observed between the PKC inhibitor treatments, further supporting the hypothesis that the effects of RSV on Akt and ERK1/2 are regulated by PKC activation.

**Figure 7:**
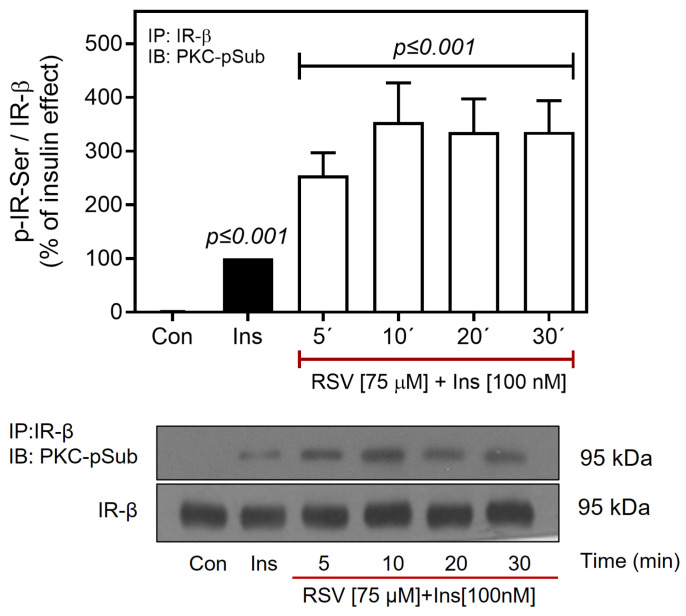
RSV promotes IR-Ser-phosphorylation through PKC activation. C9 cells were treated with or without RSV (75 µM) for 5–30 min and then stimulated with 100 nM insulin for an additional 10 min. Under all experimental conditions, untreated cells were used as controls. Total cell lysates were immunoprecipitated with an IR antibody before SDS-PAGE analysis and immunoblotted with anti-phospho-Ser-PKC substrate antibody, as described in Materials and Methods. Western blots were also probed for total IR-β showing equal loading. Data represent the mean ± S.E.M. of five to six individual experiments, and the panels below show representative immunoblots. Vertical lines represent the S.E.M. The *p*-value indicates Ins vs. control; RSV (5–30 min) + Ins [100 nM] vs. Ins.

We also determined the RSV-induced phosphorylation of IR by PKC, using a specific antibody against the phospho-(Ser) PKC substrate. Therefore, we analyzed IR immunoprecipitates for the presence of phospho-(Ser) PKC substrate motifs. As shown in Figure 7, RSV induced an increase in the PKC-mediated Ser phosphorylation of IR at 5 min (~2.5–fold increase over basal), reaching a maximum at 10 min (~3–fold increase over baseline), which persisted over the next 30 min. Taken together, these findings suggest that RSV prevents Akt activation in C9 cells by enhancing the phosphorylation of IR at Ser residues, a mechanism mediated by classical PKC isoforms. Furthermore, RSV-mediated PKC activation appears to be involved in the activation of ERK1/2. Observations in H1-6 further suggest that additional mechanisms contribute to Akt inhibition, which might operate independently of PKC, including the action of phosphatases or downstream regulators of IR.

### 2.5. Resveratrol Promotes the Activation of PTP1B

**Figure 8:**
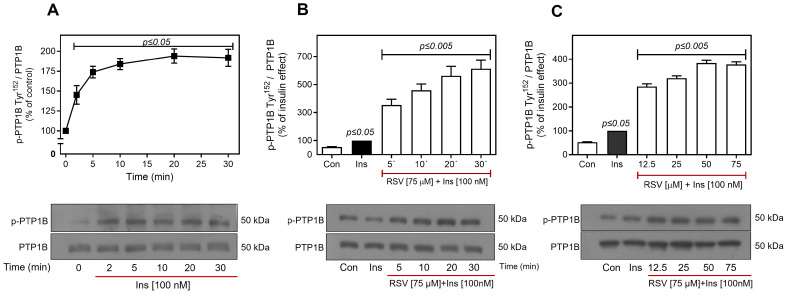
Activation of PTP1B by RSV. C9 cells were treated with 100 nM insulin for 2–30 min (**A**), pretreated with RSV (75 µM) for 5–30 min, followed by stimulation with 100 nM insulin for an additional 10 min (**B**), or pretreated with different concentrations of RSV (12.5–75 µM as indicated) for 30 min, and then stimulated with 100 nM insulin for an additional 10 min (**C**). Under all experimental conditions, untreated cells were used as controls. Total cell lysates were separated by SD-PAGE and analyzed by immunoblotting with anti-p-PTP1B-Tyr^152^ (**A**–**C**), as described in Materials and Methods. Western blots were also probed for total PTP1B showing equal loading. Data represent the mean ± S.E.M. of five to six individual experiments, and the panels below show representative immunoblots. Vertical lines represent the S.E.M. The *p*-value indicates Ins vs. control (baseline in 0 min) (**A**); Ins vs. Con (**B**,**C**); RSV (5–30 min) + Ins [100 nM] vs. Ins (**B**,**C**).

Phosphorylation of IR or IRS at Ser residues causes their dissociation from downstream substrates [76,87,88]. In contrast, dephosphorylation of Tyr residues is necessary for the inhibition of IR kinase activity, where PTPs play a critical role in the regulation of the insulin pathway. Specifically, PTP1B dephosphorylates IR and IRS, significantly contributing to the negative regulation of insulin signaling, and overactivation of PTP1B is one of the mechanisms altered in InsR and T2D [89,90]. Consequently, we investigated the effect of RSV on the activation of PTP1B. The cells were stimulated with 100 nM insulin, and the phosphorylation of PTP1B at Tyr^152^ was evaluated over 0–30 min. Insulin alone induced the phosphorylation of PTP1B, showing a significant increase after 5 min, which persisted for at least 30 min (~2–fold increase over the control) (Figure 8A). Compared to insulin, RSV induced a more pronounced increase in PTP1B phosphorylation after 5 min (~4–fold increase compared to the insulin effect), achieving a maximum at 30 min (~6–fold increase over the insulin effect) (Figure 8B). Furthermore, we observed a concentration-dependent effect of RSV, reaching a maximum effect (~4–fold increase over the insulin effect) at 50–75 µM RSV (Figure 8C). Early studies have demonstrated that PTP1B selectively dephosphorylates Tyr residues adjacent to phosphorylated Ser/Thr residues [91]. It has been proposed that PKC-mediated Ser/Thr phosphorylation of IR may enhance the subsequent interaction between PTP1B and IR, leading to significant inhibition of IR function. These findings suggest that RSV inhibits the insulin signaling pathway through a synergistic mechanism that is dependent on the activation of PKC and PTP1B. This process affects insulin signaling at the IR level and may also affect other downstream proteins, such as Akt and phosphatases, which negatively regulate insulin signaling.

## 3. Discussion

RSV, a polyphenolic compound found in significant quantities in grapes and red wine, is the subject of extensive research because of its potential health benefits, particularly in improving health and survival [92]. It has been used as a dietary supplement to improve glycemic control and reduce oxidative stress in individuals with diabetes. However, clinical trials are limited, and findings from animal or in vitro models have shown conflicting effects, such as inhibition of the insulin-induced PI3K/Akt signaling pathway in metabolic cells, including hepatic and muscle cells [35,36]. In the present study, we investigated the effects of RSV on insulin signaling in hepatic cells, focusing on C9 and H1-6 cells, both of which express endogenous IRs. These cell lines have been used as models to investigate insulin signaling in hepatic cells [41,48,55].

Initially, we characterized the response of hepatic cells to insulin by investigating the metabolic and proliferative pathways it triggers. Our results indicate that C9 and H1-6 cells respond to insulin stimulation by phosphorylating the major proteins IR/IRS/Akt and ERK1/2 (Figure 1). C9 cells showed a faster and more sustained activation of proteins involved in the insulin metabolic pathway (IR/IRS/Akt) and a reduced response to ERK1/2. Conversely, H1-6 cells exhibited a rapid response in the phosphorylation of ERK1/2, which was temporarily out of phase with IR activation. This phenomenon may result from the activation of insulin-like growth factor 1 receptors (IGF-1Rs), which are overexpressed in cancer cells and trigger the MAPK pathway [93,94,95]. Furthermore, this observation may be attributed to the constitutive activity of ERK1/2, as previously reported in these cells [55,96,97,98].

Next, we examined the effect of RSV on insulin signaling pathways in these hepatic cell models. Our findings, for the first time, demonstrate that RSV induces the concentration-dependent inhibition of insulin-stimulated IR Tyr phosphorylation, which in turn affects downstream signaling proteins, such as Akt and GS (Figure 2, Figure 3, Figure 4 and Figure 5). The maximum inhibitory concentrations of IR and Akt were comparable (Supplementary Figure S2A), which is consistent with previous reports on RSV’s effect on PI3K inhibition [36,64,72]. Statistical analysis revealed no significant differences in the IC_50_ values for either protein in C9 cells, indicating that the inhibition of Akt by RSV occurs upstream at the level of IR. Therefore, we hypothesized that in C9 cells, RSV would similarly affect the phosphorylation of IRS-1 Tyr^628^, as previous studies have indicated that the phosphorylation of Tyr^608^ and Tyr^628^ in IRS-1 is necessary for the complete activation and translocation of GLUT4 in adipocytes [38,77]. Our findings unexpectedly revealed that rather than decreasing insulin-induced IRS Tyr phosphorylation, RSV promoted an increase in IRS-1 Tyr^628^ phosphorylation (Figure 2B and Figure 4B). This suggests that IRS-1 Tyr^628^ phosphorylation may not be critical for PI3K activation or could play a secondary role in hepatic cells. This finding, along with the observation that RSV treatment reduces Tyr^1158^ IR and Ser^473^ Akt phosphorylation, suggests that PI3K activation is not entirely dependent on IRS-1 and that alternative regulatory mechanisms may exist. Early research has shown that PI3K can be activated through direct interaction with IR [99]. In vitro assays have demonstrated that the Y(P)XXM motif, particularly the Y^1322^THM motif located at the C-terminus of IR, interacts with and activates PI3K [99,100]. The Y^1322^THM motif is similar to the YXXM motif found in other PI3K-binding proteins and peptides, including IRS-1 [101]. In this regard, the deletion of part of the carboxyl-terminal sequence of IR, which contains the Y^1322^THM motif, results in diminished binding affinity of IR for the SH2 domain of PI3K [99,102].

A significant finding of the present study was that RSV inhibited insulin-induced IR and Akt activity at short prestimulation times, although in different ways. Specifically, RSV only partially inhibited IR phosphorylation (~70%) but nearly completely inhibited Akt Ser^473^ phosphorylation (~95%) at 30 min in C9 cells (Figure 3A and Figure 3B, respectively). These inconsistencies may be attributed to the mechanisms through which RSV inhibits both IR and Akt. The almost complete inhibition of Akt induced by RSV may result from inhibition at the IR level and direct RSV inhibition of PI3K, as reported by Fröjdö et al. [36].

A notable difference was observed in the effects of RSV on insulin-induced IR, Akt, and ERK activation in H1-6 cells vs. C9 cells (Figure 4A,C,D). RSV partially inhibited IR Tyr phosphorylation (~50%), whereas it almost completely inhibited Akt (~80%) and reduced ERK phosphorylation to below baseline levels within the same concentration range (Supplementary Figure S2B). A similar explanation could help clarify the results observed in the context of Akt inhibition compared to the partial inhibition of IR by RSV. This can be attributed to the direct inhibition of the PI3K/Akt pathway by RSV, as previously reported [36,72], in addition to elevated PI3K expression and activity in H1-6 hepatoma cells [96,103,104,105,106]. Furthermore, since Akt activates ERK1/2 through the PKC–Raf signaling axis [107], inactivation of the PI3K/Akt pathway by RSV may also be associated with the observed inhibition of ERK1/2 in H1-6 cells [58,61].

In C9 cells, RSV differentially reduced the insulin-stimulated phosphorylation of Akt at Ser^473^ and Thr^308^ (Figure 2C vs. Figure 5A), as indicated by the decrease in Thr^308^ phosphorylation below baseline levels. These differences may result from the RSV-induced downregulation of PDK1, which specifically phosphorylates Akt at Thr^308^, or from the RSV-mediated activation of Ser/Thr phosphatases, such as PP2A, which has a major impact on Thr^308^ phosphorylation, while affecting Ser^473^ to a lesser extent [108,109]. Early research has explored the impact of RSV and other structurally related polyphenols, demonstrating a decrease in the phosphorylation at Thr^308^ of Akt [73], which is consistent with the observations in the present study. Since RSV also activates PP2A [110] and negatively regulates PDK1 activity [111], we suggest that it mediates Akt inhibition through multiple mechanisms, including inhibition of IR, PI3K, Akt [36,72,112], or PDK1, along with activation of PP2A.

Akt-mediated insulin signaling in liver cells is essential for promoting glycogen synthesis, reducing hepatic glucose production, and promoting lipid synthesis [113]. Under normal physiological conditions, insulin stimulation leads Akt to inhibit GSK3α/β, a kinase with constitutive activity that suppresses GS [114,115]. Inhibition of IR/Akt by RSV also led to decreased phosphorylation of GSK3 (Figure 5B), indicating that GSK3α/β remains active in its function of inhibiting GS. Additionally, our results revealed differences in the decrease in phosphorylation between the two GSK3 isoforms, with a more pronounced decrease observed in the GSK3α isoform (Supplementary Figure S3). This finding is significant because, although the two isoforms share a conserved catalytic domain with 98% similarity, their activities are not redundant, and GSKs regulate processes beyond GS inhibition [116,117,118].

In this context, GSK3α is more strongly associated with insulin sensitivity and increased hepatic glycogen storage, as reported in *GSK3A* knockout (KO) mice, which exhibited elevated insulin sensitivity and increased hepatic glycogen storage [119,120]. Conversely, GSK3β regulates processes such as hepatic inflammation and apoptosis [121,122,123]. Inhibition of GS was evidenced by the increased phosphorylation of Ser^641^ observed in all RSV treatments (Figure 5C), suggesting that RSV affects glycogen synthesis in hepatic cells. This process is crucial for glucose homeostasis, as the liver removes up to one-third of the glucose load by converting it to liver glycogen [118,124]. Furthermore, deficient glycogen synthesis contributes to increased hepatic steatosis because excess carbohydrates are converted to fatty acids via de novo lipogenesis [118].

Our observations indicate that RSV exhibits heightened sensitivity to IR and Akt kinase inhibition in C9 cells compared with hepatoma cells (Supplementary Figure S2). These differences may be attributed to additional mechanisms present in C9 cells, including the direct inhibition of PI3K by RSV, which is consistent with previous reports [36]. The effect of RSV observed in our study was both concentration- and context-dependent. In this context, the inhibitory effect of RSV was concentration-dependent in all cases on IR, Akt, and GSK3, as well as on the increase or inhibition of ERK1/2 phosphorylation in C9 and H1-6 cells, respectively. Furthermore, we observed distinct cellular responses to RSV on IR and ERK in C9 and H1-6 cells. Specifically, we found that in H1-6 cells, a higher concentration of RSV was required to decrease IR phosphorylation compared to that in C9 cells. Furthermore, RSV decreased ERK1/2 phosphorylation in H1-6 cells, whereas in C9 cells, it promoted their phosphorylation. The latter clearly indicates a differential effect of RSV, depending on the cellular context.

To further elucidate how RSV downregulates IR Tyr^1158^ phosphorylation, we investigated two well-established mechanisms of negative regulation of the insulin pathway: phosphorylation of IR/IRS by PKCs [41,77] and dephosphorylation of IR by PTP1B [90,125]. The PKC family is classified into three groups: classical (α, βI, βII, and γ), novel (δ, ε, η and θ), and atypical (ζ and λ/τ) [126,127], all of which play critical roles as mediators of insulin signaling [78,82,84,128,129]. Among the classical PKCs, PKCα has been identified to play a significant role in the inhibition of IR activity and activation of PI3K by IRS-1. KO mice of PKCα show improved enzymatic activity of IR and its downstream target proteins (PI3K, Akt, and ERK) in muscle and adipocytes [129,130]. With respect to novel PKCs, PKCε has been reported to negatively regulate IR kinase activity, IRS phosphorylation, insulin clearance, and IR degradation [131], particularly in hepatic tissue. Elevated levels of PKCε have been observed in the liver of rats with non-insulin-dependent diabetes mellitus (NIDDM) and in patients with T2D [132]. Experimental studies using antisense oligonucleotides against PKCε in rats demonstrated that PKCε inhibition reverses IR kinase defects and restores glucose tolerance [79,131]. Therefore, this study investigated the effects of inhibiting both classical and novel PKC activities in a global context using specific inhibitors [77]. PKC inhibition by BIM and Gö6976 prevented the RSV-induced decrease in Akt phosphorylation in C9 (Figure 6A) and H1-6 cells (Figure 6B). Moreover, PKC inhibition recovered the effect of RSV on GSK3 phosphorylation (Supplementary Figure S4C), thereby maintaining active glycogen synthesis. As both PKC inhibitors inhibited the RSV effect equally, our results suggest that classical PKC isoenzymes, PKCα and PKCβ, participate in the actions of RSV.

Regarding how RSV differentially affects insulin-induced ERK1/2 phosphorylation in C9 and H1-6 cells, the use of the PKC inhibitors BIM and Gö6976 reversed the increase in ERK1/2 phosphorylation caused by RSV in C9 cells (Figure 6C) and restored its inhibitory effect in H1-6 cells (Figure 6D). Although our findings indicate that the classical PKCα and PKCβI isoforms modulate RSV’s effects, this does not elucidate the activation of ERK1/2 in C9 cells or its inhibition in H1-6 cells, where ERK1/2 is more sensitive to RSV concentration than IR. This suggests that RSV regulation of ERK1/2, at least in C9 cells, may occur downstream of the receptor, possibly mediated by the same PKCs, either by acting directly on ERK1/2 or by influencing intermediate proteins in the pathway. In this regard, specific PKC isoforms are known to activate the ERK1/2 pathway. For instance, PKCα—but not PKCβII or PKCε—mediates Ras and Raf-independent MEK/ERK1/2 activation in phorbol ester-stimulated HepG2 cells [133,134]. Similarly, in Chinese hamster ovary cells, PKCα was found to be involved in αVβ3 integrin-mediated ERK1/2 activation in an intracellular Ca^2+^-dependent manner but not through the classical Shc/Ras/Raf-1 pathway [135]. In contrast, in endothelial cells, Raf-1/ERK1/2 activation is triggered by the sequential activation of PKCα and PKCε, along with their temporal association with Raf-1; however, other PKC isoforms may also be involved [136]. Likewise, PKCβ was found to play a critical role as a modulator of hepatocellular carcinoma cell motility and invasion through the activation of the ERK1/2/p38MAPK-HSP27 pathway [137]. Additionally, phorbol ester-induced PKCβ activation was found to activate ERK1/2, which is associated with the expression of neuronal differentiation genes in neuroblastoma cells [138]. Concerning the inhibitory effect of RSV on insulin-induced ERK1/2 activation observed in H1-6 cells, this may be related to PKC’s inhibition of IR itself, which affects the classic ERK1/2 activation pathway described for IR (IR/Shc/GRB2/SOS/Ras/RAF/MEK/ERK1/2) [37]. Nonetheless, it cannot be ruled out that PKC also inhibits the downstream signaling of IR.

Multiple studies have demonstrated the effect of PKC on the negative regulation of the insulin pathway. For instance, PKC isoenzymes α, ε and ζ, were found to be significantly increased in the membrane fractions of liver biopsies from patients with obesity and NIDDM, as well as in Zucker diabetic fatty rats, when compared to controls [132]. Although no alterations in PKCβ were detected in the liver of patients, an increase in this isoform was observed in the rat model compared to the controls [132]. Furthermore, in rat fibroblasts stably overexpressing IRs, it was shown that stimulation with glucose or phorbol esters induces the translocation of PKCα, δ, ε, and ζ isoforms to the plasma membrane and significantly reduces IR kinase activity and Tyr phosphorylation of IRS [139]. Consistent with this, the kinetics of IR inhibition correlated with PKC translocation to the plasma membrane, in addition to its inhibition by H-7, which prevented the effect of glucose on IR, demonstrating that PKC negatively regulates IR/IRS activity [139].

Other studies have demonstrated that PKCα plays a key role in regulating insulin signaling, reducing the activity of IR and/or IRS-1 by phosphorylating Ser residues, which affects its ability to regulate downstream substrates to propagate the signal [77,140,141]. In this context, although our results on IRS suggest that RSV does not cause a decrease in Tyr phosphorylation associated with signaling desensitization, they cannot rule out that RSV can regulate insulin signaling at the level of the IR through a PKC-mediated mechanism. To evaluate whether RSV regulates inhibition of the insulin pathway at the IR level, we immunoprecipitated IR and detected PKC-induced IR phosphorylation using a phospho-(Ser)-PKC substrate motif antibody (Figure 7). RSV induced IR phosphorylation via PKC in a time-dependent manner, suggesting that RSV could directly regulate insulin signaling by phosphorylating IR at Ser residues, which is a PKC-mediated mechanism.

Phosphorylation of the receptor on Ser/Thr residues may regulate its uncoupling from the IRS. However, considering the decrease in IR Tyr^1158^ phosphorylation, we investigated whether this effect was dependent on the activity of phosphatases, such as PTP1B. PTP1B is recognized as an important regulator of IR, and KO models of PTP1B or the use of antisense oligonucleotides of this protein show improvement in insulin sensitivity and normalization of blood glucose, effects that have been attributed to increased phosphorylation of IR in the liver and muscle [142,143,144,145]. In this study, we demonstrated that insulin stimulation induced a significant increase in PTP1B Tyr^152^ phosphorylation from 2 min, which remained constant until 30 min of insulin stimulation. However, notably, RSV was able to elicit a significant increase in Tyr^152^ phosphorylation of PTP1B, which depended on time and concentration (Figure 8), and has been associated with the activation of the phosphatase and its interaction with IR [146,147]. Our observations indicate that RSV can activate PTP1B, and the observed reduction in IR phosphorylation may be attributed to the action of this protein. Previous research has demonstrated the specificity of PTP1B in interacting with regions of the IR where Tyr-phosphorylated residues are flanked by amino acids such as Ser, Thr, Glu, and Asp, or are located near tandem Tyr phosphorylation [56,91]. Therefore, we propose that phosphorylation of IR by PKC may also facilitate its interaction with active PTP1B.

Our findings describe a mechanism by which RSV inhibits the signaling pathway in liver cells by activating PKC and PTP1B. However, the precise mechanism by which RSV regulates these proteins remains uncertain, as the mechanisms of action of RSV vary among cell types, and some are controversial. Nevertheless, it is currently recognized that both phosphatases and kinases are the targets of redox regulation. Based on the literature regarding the antioxidant effects of RSV, it can be inferred that the activation of these proteins by RSV relies on oxidative modifications [148,149,150,151]. In redox regulation, the presence of oxidation-sensitive amino acids is critical for protein activation or inhibition. Specifically, the oxidation of aromatic residues, such as Tyr, Trp, and Phe, induces conformational changes that render the protein receptive to interactions with inhibitors or agonists [149,152]. Furthermore, sulfur-containing amino acids, such as Cys and Met, are more susceptible to oxidation. Because this oxidation is reversible, it plays a significant role in cellular signaling [149,153].

Research indicates that the oxidation of the Cys residue at the catalytic site in PTPs, mediated by ROS, particularly Cys^215^ of PTP1B, leads to enzymatic inactivation. Consequently, inhibitors or antioxidants that reduce ROS levels can prevent the oxidation of phosphatases [150,154,155,156]. Conversely, oxidative modifications have also been identified in kinases, such as the oxidation of Src at Cys^245^ and Cys^487^ [157,158,159], which enhances its activity, and the formation of disulfide bonds between Cys^124^, Cys^297^, and Cys^311^ of Akt, which leads to activation or inactivation, depending on the isoform [160,161,162]. In this type of regulation, ROS concentrations can produce different effects at high and low concentrations. For instance, ERK2 can be directly oxidized into two Cys residues at low concentrations of H_2_O_2_ (0.1 µM) but not when exposed to higher concentrations (>10 µM) [163]. Redox regulation of PKC has been described, involving the generation of diacylglycerol and Ca^2+^ release through RTK-activated PLCγ and direct oxidation of PKC Cys residues [164,165]. As PKC isoforms contain zinc fingers and numerous Cys residues in their regulatory domains, they are susceptible to oxidation, which increases or inhibits their activity depending on the oxidation site [151,166,167].

Finally, another potential regulatory point downstream of IR/IRS by RSV is at the level of Akt. Considering that our findings support a key role of PKC in the regulatory effects of RSV, they are consistent with previous reports demonstrating the significant role of PKC in the negative regulation of insulin, particularly PKCα, at different key levels in the signaling pathway, such as the activation of PP2A. The data obtained from our study suggest that classical PKC isoforms are responsible for inhibiting the PI3K/Akt pathway by RSV, consistent with previous work indicating that activation of both classical and novel PKC isoforms leads to the dephosphorylation of Akt through a mechanism that involves the activation of PP2A [78,168]. An interesting finding indicates that in patient samples, animal models, and human endometrial cancer cell lines, PKCα exhibited a key tumor-suppressive role mediated by its ability to suppress the PI3K/Akt signaling pathway by activating PP2A [168,169]. However, we cannot exclude the possibility that novel PKC isoforms contribute to the effects of RSV, especially PKCε, which is predominant in the liver and is associated with hepatic InsR, in addition to causing PKCα-like effects on IR and PP2A [78,79,83].

Regarding the effects observed by RSV in other metabolic tissues and in our study, the inhibition of the PI3K/Akt pathway in C9 and H1-6 cells confirmed previous findings in the liver [35,36], muscle [32], and adipose tissue [29]. However, given the tissue-specific effects of RSV, the beneficial effects observed in adipose tissue and muscle can be explained. For example, in adipocytes, although Akt-Ser^473^ phosphorylation is reduced, RSV also promotes an increase in IRS and GLUT4 levels [30], which could compensate for the deficiency in Akt activation, as glucose uptake may still be enhanced. The negative regulation of PI3K/Akt by ESR1 activation in adipocytes [29] may represent a relevant mechanism in the liver that explains the increased phosphorylation of IRS [170,171]. In contrast, previous findings in muscle are consistent with those observed in C9 cells, where RSV treatment in normal cells reduced Akt phosphorylation by up to 50% at a concentration of 50 µM. Nevertheless, glucose uptake does not appear to be affected by RSV [32]. Additionally, in insulin-resistant cells, RSV promotes the expression of phosphorylated IR and downstream proteins [32,172], suggesting that under altered metabolic conditions, RSV may exert beneficial effects on hepatocytes.

Another important finding of this study is the regulation of GSK3. Previously, in rats fed a HFSD, RSV increased the phosphorylation of GSK3β [33]; however, in C9 cells, it caused a decrease in both isoforms, with GSK3α being more affected. This isoform is associated with hepatic glycogen storage. Although the inhibition of the PI3K/Akt pathway has also been reported in muscle and adipose tissue, compensatory mechanisms appear to exist that positively regulate upstream proteins, such as IR/IRS, and downstream targets, such as GLUT4 [29,30,172]. However, the findings in hepatic cells indicated almost complete inhibition of the pathway from IR to GSK3 and GS proteins, resulting in decreased glycogen synthesis. It is important to evaluate whether the RSV-induced increase in IRS phosphorylation represents a beneficial effect in the liver.

**Figure 9:**
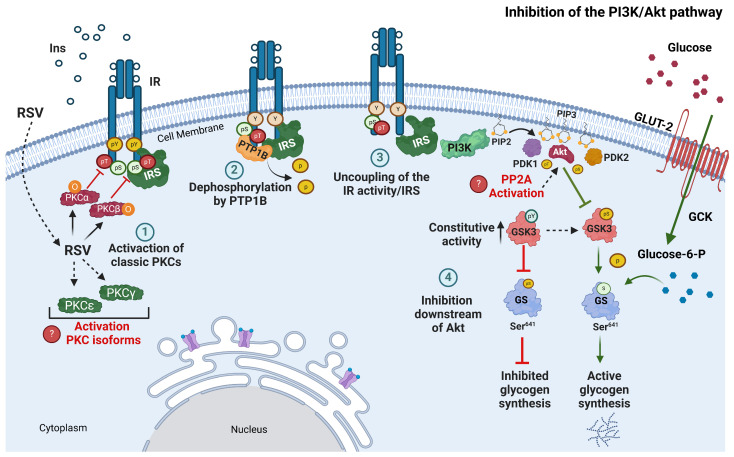
Model of desensitization of insulin signaling pathway by RSV in C9 cells. When hepatic cells are treated with RSV, classical PKC isoforms are activated, which in turn interact with the insulin receptor (IR) and phosphorylate it on serine residues (Ser). Subsequently, PTP1B dephosphorylates the receptor and causes uncoupling with IRS, impairing insulin actions, such as hepatic glycogen synthesis. The numbered circles indicate the process of inhibition of the PI3K/Akt pathway by RSV: (1) RSV activates classic isoforms of PKC that phosphorylate Ser/Thr residues in IR; (2) previous phosphorylation of Ser/Thr in IR facilitates the interaction of PTP1B with IR and dephosphorylates it; (3) inhibition of IR causes uncoupling of IRS; and (4) downstream of the PI3K/Akt pathway is inhibited. Green arrows indicate normal insulin regulation, and red arrows indicate the inhibition of RSV-regulated processes. The diagram indicates hypothetical processes in red letters that could contribute to the negative regulation of the PI3K/Akt pathway, such as the activation of other PKC isoforms and the PP2A phosphatase. Ins, insulin; GCK, glucokinase; pSer, serine phosphorylation; pThr, threonine phosphorylation; pTyr, tyrosine phosphorylation; RSV, resveratrol. Figure created using BioRender.com (2024).

In summary, the present findings indicate that, in hepatic C9 and H1-6 cells, RSV modulates the activation of the insulin-stimulated PI3K/Akt pathway through a mechanism dependent on the activation of classical PKC isoforms and PTP1B (Figure 9). Additionally, our results suggest that RSV can regulate ERK1/2 activation or inhibition by activating classical PKCα and PKCβI isoforms (Supplementary Figure S5). However, the potential involvement of other isoforms, such as PKCγ, and novel PKCs, including PKCδ may not be excluded.

These findings provide new insights into the role of RSV in hepatic cells and its potential adverse effects on the liver when utilized as a supplement to treat InsR and other related liver metabolic disorders, such as metabolic dysfunction-associated steatotic liver disease (MASLD) [173]. Previous research has demonstrated that inhibiting proteins in the canonical insulin pathway, IR/IRS/PI3K/Akt [173,174,175], in the liver also suppresses *de novo* lipogenesis (DNL) at InsR. However, the inhibition of proteins such as mTORC1 and SREBP-1c, which are downstream of Akt and may partially regulate DNL, is insufficient to mediate this process [176,177]. This suggests that the altered insulin pathway continues to regulate increased DNL until the development of MASLD, although the extent of its contribution remains unknown. Furthermore, mTORC1 and SREBP-1c activity are known to remain somewhat independent of Akt (possibly regulated by IRS or independent of the canonical pathway) [173], which could contribute to the development of hepatic steatosis. Other studies have also indicated that hepatic lipid accumulation results not only from hepatic DNL but also from extrahepatic factors, such as circulating FFAs [178,179,180]. This suggests that RSV-induced InsR could mediate other hepatic alterations through specific effects on different metabolic tissues, such as muscle or adipose tissue. This necessitates the use of disease models to explore how RSV influences insulin function in energy metabolism and whether it also influences liver dysfunction.

## 4. Materials and Methods

### 4.1. Reagents, Peptides, Inhibitors, and Antibodies

Ham’s F12K nutrient mixture, Kaigh’s modification (F12 K medium), Dulbecco’s modified Eagle’s medium (DMEM) high glucose (4500 mg/L), with L-glutamine (4 mM), sodium pyruvate (1 Mm), and 1500 mg/L sodium bicarbonate, resveratrol, and reagents for electrophoresis were obtained from Sigma-Aldrich (St. Louis, MO, USA). Fetal bovine serum (FBS), antibiotic-antimycotic 100 x (penicillin, streptomycin, and amphotericin B), and bovine insulin were purchased from Invitrogen (Carlsbad, CA, USA). Trypsin-EDTA 0.25% was obtained from Thermo Fisher Scientific (Waltham, MA, USA). BIM, Gö6976, and protease inhibitor cocktail Set III were obtained from Calbiochem (La Jolla, CA, USA). The enhanced chemiluminescence (ECL) reagent was obtained from Immobilon^TM^ Millipore Corporation (Billerica, MA, USA). The primary and secondary antibodies used for Western blotting are listed in Supplementary Table S1.

### 4.2. Cell Culture and Experiments

C9 and H1-6 cells were obtained from the American Type Culture Collection (ATCC, Manassas, VA, USA) and cultured in F12K medium or DMEM supplemented with 10% (*v*/*v*) FBS (non-heat-inactivated), 100 IU/mL penicillin, 100 µg/mL streptomycin, and 250 ng/mL amphotericin B at 37 °C in humidified O_2_ (95%)/CO_2_ (5%). C9 and H1-6 cells were used between passages 2 and 12, when they exhibited a clear response to insulin stimulation [41,48,55,181]. These cells were then reseeded onto six- or twelve-well plates until they reached 80% confluence. Six hours before each experiment, C9 and H1-6 hepatic cells were switched to serum-free medium and treated with the indicated ligands and inhibitors.

For acute insulin stimulation in cell cultures, typical pharmacological insulin concentrations range from 0.1 nM to 1000 nM, with 100 nM often being the highest stimulating concentration [49,182,183]. In previous studies from our group, particularly with hepatic C9 cells, we have used 100 nM as the highest stimulus [41,48,77,182].

The RSV concentrations employed in our study, ranging from 12.5 µM to 100 µM, are justified for in vitro experiments with liver cells due to their demonstrated biological relevance [35,36,64,184,185]. This range facilitates the exploration of dose-dependent effects and is physiologically relevant because the liver, the main site of resveratrol metabolism, experiences higher local concentrations than peripheral tissues [36,186,187,188]. Furthermore, these concentrations are consistent with those successfully used in existing in vitro studies, ensuring their comparability. The inhibitor concentrations were based on the IC_50_ values reported by the manufacturer and in previous reports from our group [77,189].

C9 and H1-6 cells were stimulated with 100 nM insulin for 0–30 min to determine the activation status of the insulin pathway. After confirming the insulin response in both cell types, assays were performed to investigate the effects of RSV on insulin signaling. C9 and H1-6 cells were pretreated for 30 min with increasing concentrations of RSV (ranging from 12.5 to 75 μM) or (from 12.5 to 100 μM), respectively, and subsequently stimulated with 100 nM insulin for 10 min. To evaluate the time-dependent effect of RSV, C9 cells were pretreated with RSV (75 µM) for 5, 10, 20, and 30 min and then stimulated with 100 nM insulin for an additional 10 min. Untreated cells were used as controls under all experimental conditions. To investigate the possible role of PKC in RSV-mediated downregulation of the insulin pathway, cells were pretreated with 1 µM BIM [77], or 100 nM Gö6976 [85,86] for 30 min, followed by RSV (75 µM) treatment for 30 min and 100 nM insulin stimulation for 10 min.

### 4.3. Western Blot Methods

After treatment, cells were placed on ice, the media was aspirated, and the cells were washed twice with ice-cold PBS and lysed in 100 µL RIPA sample buffer 1X. The samples were briefly sonicated, heated at 99 °C, and centrifuged for 5 min at 14,000 rpm. The resulting supernatants were loaded into separate lanes of SDS-PAGE (6%, 8%, or 10%) gels, electrophoresed, and transferred to polyvinylidene difluoride membranes (Millipore, Billerica, MA, USA). Blocking was carried out by incubation for 1 h with 5% Blotto, non-fat dry milk, or 3% bovine serum albumin (BSA) (Santa Cruz Biotechnology, Inc., Santa Cruz, CA, USA) in TBS/0.1% Tween (TBST) at room temperature. Western blots were then probed with specific antibodies targeting phosphorylated and non-phosphorylated forms of IR, IRS-1, Akt, ERK1/2, and PKC isoforms for primary immunodetection. The other primary antibodies used in this study, along with their corresponding dilutions, are listed in Supplementary Table S1. The membranes were incubated overnight at 4 °C with primary antibodies, washed three times with TBST, and probed with horseradish peroxidase-conjugated secondary antibodies for 1 or 2 h at room temperature. Protein bands were visualized using an enhanced chemiluminescence ECL reagent and scanned.

### 4.4. Immunoprecipitation Assay for Ser/Thr Phosphorylated IR

C9 hepatic cells were grown in 10 cm dishes and serum-starved for 6 h before treatment with 75 µM RSV and 100 nM insulin for the indicated times at 37 °C. The cells were washed twice with ice-cold PBS and lysed in Nonidet-P-40 solubilization buffer (50 mM Tris-HCl, 150 mM NaCl, 2 mM orthovanadate, 1 mM NaF, 1% Nonidet P–40, 10% glycerol, and 2 mM EDTA, pH 7.4) containing protease and phosphatase inhibitors. After immunoprecipitation of IR with anti-IR-β polyclonal antibody (Santa Cruz Biotechnology, Santa Cruz, CA, USA) and protein A/G PLUS-Agarose (Santa Cruz Biotechnology, Santa Cruz, CA, USA), the proteins were resolved by SDS-PAGE, Western-blotted, and probed with phospho-PKC-substrate antibody (Cell Signaling Technology, Danvers, MA, USA), which detects proteins containing phospho-Ser residues in the motif (R/K)X(pS)(Hyd)(R/K), where Hyd is any hydrophobic amino acid and X is any amino acid, followed by a horseradish peroxidase conjugate to identify phosphorylated proteins. Blots were visualized and quantified as previously described.

### 4.5. Statistical Analysis

The average intensities from Western blotting were analyzed using either one- or two-way ANOVA. If statistical significance was found, planned post hoc analyses were performed using Dunnett’s test (comparison to control) or Bonferroni’s multiple comparison test to determine individual group differences using PRISM, Version 10.4.1 (GraphPad Software, San Diego, CA, USA). In all cases, *p* < 0.05 was considered to be significant. Data were normalized using either the control or insulin responses, and the mean ± S.E.M. was plotted for at least five separate experiments. Figures show representative blots. Normalized densitometry values (phosphorylated protein/actin) are presented in Supplementary Tables S2–S9. Values were rounded to three decimal places.

## 5. Conclusions

In conclusion, our findings indicate that RSV alters insulin-induced IR/Akt/GSK3/GS signaling through a mechanism dependent on classical PKC and PTP1B activation. The PKC-mediated phosphorylation of IR favors the interaction between PTP1B and IR. It is possible that the activation of PKCs and PTP1B is mediated by oxidative modifications in the regulatory or catalytic domains of each protein, which are affected by the antioxidant activity of RSV. It is necessary to evaluate the direct effects of RSV on specific isoforms of PKC and the phosphorylation of PTP1B to elucidate the mechanism of activation of both proteins.

## Data Availability

The data presented in this study are available upon request from the corresponding author.

## Supplementary Materials

The following supporting information can be downloaded at: https://www.mdpi.com/article/10.3390/ijms26157434/s1.

## Abbreviations

BIM	Bisindolylmaleimide I
C9 cells	Clone 9 cells
CAT	Catalases
DNL	De novo lipogenesis
FFA	Free fatty acid
GLUT4	Glucose transporter 4
GPX	Glutathione peroxidase
GS	Glycogen synthase
GSK3α/β	Glycogen synthase kinase-3 α/β
H1-6	Hepa 1-6 cells
HFSD	High-fat/high-sugar diet
InsR	Insulin resistance
IR	Insulin receptor
IRS	Insulin receptor substrate
MASLD	Metabolic dysfunction-associated steatotic liver disease
NIDDM	Non-insulin-dependent diabetes mellitus
PI3K	Phosphatidylinositol 3-kinase
PKC	Protein kinase C
PTP	Protein tyrosine phosphatase
PTP1B	Protein tyrosine phosphatase 1B
ROS	Reactive oxygen species
RSV	Resveratrol
Ser	Serine
SOD	Superoxide dismutase
T2D	Type 2 diabetes
Thr	Threonine
Tyr	Tyrosine

## References

[1] Li Z., Chen X., Liu G., Li J., Zhang J., Cao Y., Miao J. (2021). Antioxidant Activity and Mechanism of Resveratrol and Polydatin Isolated from Mulberry (*Morus Alba* L.). Molecules.

[2] Akinwumi B., Bordun K.-A., Anderson H. (2018). Biological Activities of Stilbenoids. Int. J. Mol. Sci..

[3] Salehi B., Mishra A.P., Nigam M., Sener B., Kilic M., Sharifi-Rad M., Fokou P.V.T., Martins N., Sharifi-Rad J. (2018). Resveratrol: A Double-Edged Sword in Health Benefits. Biomedicines.

[4] Tong W., Chen X., Song X., Chen Y., Jia R., Zou Y., Li L., Yin L., He C., Liang X. (2020). Resveratrol Inhibits Lps-Induced Inflammation through Suppressing the Signaling Cascades of Tlr4-Nf-κb/Mapks/Irf3. Exp. Ther. Med..

[5] Kursvietiene L., Kopustinskiene D.M., Staneviciene I., Mongirdiene A., Kubová K., Masteikova R., Bernatoniene J. (2023). Anti-Cancer Properties of Resveratrol: A Focus on Its Impact on Mitochondrial Functions. Antioxidants.

[6] Sadi G., Şahin G., Bostanci A. (2018). Modulation of Renal Insulin Signaling Pathway and Antioxidant Enzymes with Streptozotocin-Induced Diabetes: Effects of Resveratrol. Medicina.

[7] Reda D., Elshopakey G.E., Mahgoub H.A., Risha E.F., Khan A.A., Rajab B.S., El-Boshy M.E., Abdelhamid F.M. (2022). Effects of Resveratrol against Induced Metabolic Syndrome in Rats: Role of Oxidative Stress, Inflammation, and Insulin Resistance. Evid. Based Complement. Alternat. Med..

[8] Ormazabal V., Nair S., Elfeky O., Aguayo C., Salomon C., Zuñiga F.A. (2018). Association between Insulin Resistance and the Development of Cardiovascular Disease. Cardiovasc. Diabetol..

[9] Khattar S., Khan S.A., Zaidi S.A.A., Darvishikolour M., Farooq U., Naseef P.P., Kurunian M.S., Khan M.Z., Shamim A., Khan M.M.U. (2022). Resveratrol from Dietary Supplement to a Drug Candidate: An Assessment of Potential. Pharmaceuticals.

[10] Su M., Zhao W., Xu S., Weng J. (2022). Resveratrol in Treating Diabetes and Its Cardiovascular Complications: A Review of Its Mechanisms of Action. Antioxidants.

[11] Cottart C.H., Nivet-Antoine V., Laguillier-Morizot C., Beaudeux J.L. (2010). Resveratrol Bioavailability and Toxicity in Humans. Mol. Nutr. Food Res..

[12] Patel K.R., Scott E., Brown V.A., Gescher A.J., Steward W.P., Brown K. (2011). Clinical Trials of Resveratrol. Ann. N. Y. Acad. Sci..

[13] Shaito A., Posadino A.M., Younes N., Hasan H., Halabi S., Alhababi D., Al-Mohannadi A., Abdel-Rahman W.M., Eid A.H., Nasrallah G.K. (2020). Potential Adverse Effects of Resveratrol: A Literature Review. Int. J. Mol. Sci..

[14] Jhanji M., Rao C.N., Sajish M. (2021). Towards Resolving the Enigma of the Dichotomy of Resveratrol: Cis- and Trans-Resveratrol Have Opposite Effects on Tyrrs-Regulated Parp1 Activation. GeroScience.

[15] Jhanji M., Rao C.N., Massey J.C., Hope M.C., Zhou X., Keene C.D., Ma T., Wyatt M.D., Stewart J.A., Sajish M. (2022). Cis- and Trans-Resveratrol Have Opposite Effects on Histone Serine-Adp-Ribosylation and Tyrosine Induced Neurodegeneration. Nat. Commun..

[16] Komorowska D., Gajewska A., Hikisz P., Bartosz G., Rodacka A. (2021). Comparison of the Effects of Resveratrol and Its Derivatives on the Radiation Response of Mcf-7 Breast Cancer Cells. Int. J. Mol. Sci..

[17] Zheng X., Jia B., Tian X.-T., Song X., Wu M.-L., Kong Q.-Y., Li H., Liu J. (2018). Correlation of Reactive Oxygen Species Levels with Resveratrol Sensitivities of Anaplastic Thyroid Cancer Cells. Oxid. Med. Cell. Longev..

[18] Konopko A., Litwinienko G. (2022). Unexpected Role of Ph and Microenvironment on the Antioxidant and Synergistic Activity of Resveratrol in Model Micellar and Liposomal Systems. J. Org. Chem..

[19] Pignitter M., Schueller K., Burkon A., Knorr V., Esefelder L., Doberer D., Wolzt M., Somoza V. (2016). Concentration-Dependent Effects of Resveratrol and Metabolites on the Redox Status of Human Erythrocytes in Single-Dose Studies. J. Nutr. Biochem..

[20] Salami S.A., Guinguina A., Agboola J.O., Omede A.A., Agbonlahor E.M., Tayyab U. (2016). Review: In Vivo and Postmortem Effects of Feed Antioxidants in Livestock: A Review of the Implications on Authorization of Antioxidant Feed Additives. Animal.

[21] Desjardins D., Cacho-Valadez B., Liu J.-L., Wang Y., Yee C., Bernard K., Khaki A., Breton L., Hekimi S. (2017). Antioxidants Reveal an Inverted U-Shaped Dose-Response Relationship between Reactive Oxygen Species Levels and the Rate of Aging in Caenorhabditis Elegans. Aging Cell.

[22] Xia N., Daiber A., Förstermann U., Li H. (2017). Antioxidant Effects of Resveratrol in the Cardiovascular System. Br. J. Pharmacol..

[23] Brioukhanov A.L., Netrusov A.I. (2004). Catalase and Superoxide Dismutase: Distribution, Properties, and Physiological Role in Cells of Strict Anaerobes. Biochemistry.

[24] Jeyaraman M.M., Al-Yousif N.S.H., Singh Mann A., Dolinsky V.W., Rabbani R., Zarychanski R., Abou-Setta A.M. (2020). Resveratrol for Adults with Type 2 Diabetes Mellitus. Cochrane Database Syst. Rev..

[25] Walker J.M., Eckardt P., Aleman J.O., da Rosa J.C., Liang Y., Iizumi T., Etheve S., Blaser M.J., Breslow J.L., Holt P.R. (2019). The Effects of Trans-Resveratrol on Insulin Resistance, Inflammation, and Microbiota in Men with the Metabolic Syndrome: A Pilot Randomized, Placebo-Controlled Clinical Trial. J. Clin. Transl. Res..

[26] Wong R.H.X., Howe P.R.C. (2018). Resveratrol Counteracts Insulin Resistance—Potential Role of the Circulation. Nutrients.

[27] Szkudelska K., Deniziak M., Sassek M., Szkudelski I., Noskowiak W., Szkudelski T. (2021). Resveratrol Affects Insulin Signaling in Type 2 Diabetic Goto-Kakizaki Rats. Int. J. Mol. Sci..

[28] Movahed A., Raj P., Nabipour I., Mahmoodi M., Ostovar A., Kalantarhormozi M., Netticadan T. (2020). Efficacy and Safety of Resveratrol in Type 1 Diabetes Patients: A Two-Month Preliminary Exploratory Trial. Nutrients.

[29] Yang B., Wang Q., Li Y., Zhang S., Sun Y., Wei Y., Jiang Q., Huang Y. (2024). Resveratrol Inhibits White Adipose Deposition by the Esr1-Mediated Pi3k/Akt Signaling Pathway. Cell. Signal..

[30] Jimenez-Gomez Y., Mattison J.A., Pearson K.J., Martin-Montalvo A., Palacios H.H., Sossong A.M., Ward T.M., Younts C.M., Lewis K., Allard J.S. (2013). Resveratrol Improves Adipose Insulin Signaling and Reduces the Inflammatory Response in Adipose Tissue of Rhesus Monkeys on High-Fat, High-Sugar Diet. Cell Metab..

[31] Gong L., Guo S., Zou Z. (2020). Resveratrol Ameliorates Metabolic Disorders and Insulin Resistance in High-Fat Diet-Fed Mice. Life Sci..

[32] Quan Y., Hua S., Li W., Zhan M., Li Y., Lu L. (2020). Resveratrol Bidirectionally Regulates Insulin Effects in Skeletal Muscle through Alternation of Intracellular Redox Homeostasis. Life Sci..

[33] Zhao H., Zhang Y., Shu L., Song G., Ma H. (2019). Resveratrol Reduces Liver Endoplasmic Reticulum Stress and Improves Insulin Sensitivity in Vivo and in Vitro. Drug Des. Devel. Ther..

[34] Du F., Huang R., Lin D., Wang Y., Yang X., Huang X., Zheng B., Chen Z., Huang Y., Wang X. (2021). Resveratrol Improves Liver Steatosis and Insulin Resistance in Non-Alcoholic Fatty Liver Disease in Association with the Gut Microbiota. Front. Microbiol..

[35] Zhang J. (2006). Resveratrol Inhibits Insulin Responses in a Sirt1-Independent Pathway. Biochem. J..

[36] Fröjdö S., Cozzone D., Vidal H., Pirola L. (2007). Resveratrol Is a Class Ia Phosphoinositide 3-Kinase Inhibitor. Biochem. J..

[37] Saltiel A.R. (2021). Insulin Signaling in Health and Disease. J. Clin. Investig..

[38] Esposito D.L., Li Y., Cama A., Quon M.J. (2001). Tyr612 and Tyr632 in Human Insulin Receptor Substrate-1 Are Important for Full Activation of Insulin-Stimulated Phosphatidylinositol 3-Kinase Activity and Translocation of Glut4 in Adipose Cells. Endocrinology.

[39] Boucher J., Kleinridders A., Kahn C.R. (2014). Insulin Receptor Signaling in Normal and Insulin-Resistant States. Cold Spring Harb. Perspect. Biol..

[40] Najjar S.M., Perdomo G. (2019). Hepatic Insulin Clearance: Mechanism and Physiology. Physiology.

[41] Roura-Guiberna A., Hernandez-Aranda J., Ramirez-Flores C.J., Mondragon-Flores R., Garibay-Nieto N., Queipo-Garcia G., Laresgoiti-Servitje E., Soh J.-W., Olivares-Reyes J.A. (2019). Isomers of Conjugated Linoleic Acid Induce Insulin Resistance through a Mechanism Involving Activation of Protein Kinase Cε in Liver Cells. Cell. Signal..

[42] Whelan S.A., Dias W.B., Thiruneelakantapillai L., Lane M.D., Hart G.W. (2010). Regulation of Insulin Receptor Substrate 1 (Irs-1)/Akt Kinase-Mediated Insulin Signaling by O-Linked Beta-N-Acetylglucosamine in 3t3-L1 Adipocytes. J. Biol. Chem..

[43] Fayard E., Xue G., Parcellier A., Bozulic L., Hemmings B.A. (2010). Protein Kinase B (Pkb/Akt), a Key Mediator of the Pi3k Signaling Pathway. Curr. Top. Microbiol. Immunol..

[44] Lee S.H., Park S.Y., Choi C.S. (2022). Insulin Resistance: From Mechanisms to Therapeutic Strategies. Diabetes Metab. J..

[45] Rui L. (2014). Energy Metabolism in the Liver. Compr. Physiol..

[46] Ader M., Bergman R.N. (2021). Hyperinsulinemic Compensation for Insulin Resistance Occurs Independent of Elevated Glycemia in Male Dogs. Endocrinology.

[47] Weinstein I.B., Orenstein J.M., Gebert R., Kaighn M.E., Stadler U.C. (1975). Growth and Structural Properties of Epithelial Cell Cultures Established from Normal Rat Liver and Chemically Induced Hepatomas1. Cancer Res..

[48] Arellano-Plancarte A., Hernandez-Aranda J., Catt K.J., Olivares-Reyes J.A. (2010). Angiotensin-Induced Egf Receptor Transactivation Inhibits Insulin Signaling in C9 Hepatic Cells. Biochem. Pharmacol..

[49] Defries D.M., Taylor C.G., Zahradka P. (2016). Glut3 Is Present in Clone 9 Liver Cells and Translocates to the Plasma Membrane in Response to Insulin. Biochem. Biophys. Res. Commun..

[50] Quintanilla R.A., Porras O.H., Castro J., Barros L.F. (2000). Cytosolic [Ca(2+)] Modulates Basal Glut1 Activity and Plays a Permissive Role in Its Activation by Metabolic Stress and Insulin in Rat Epithelial Cells. Cell Calcium.

[51] Shetty M., Kuruvilla A.K., Ismail-Beigi F., Loeb J.N. (1996). Stimulation of Glucose Transport in Clone 9 Cells by Insulin and Thyroid Hormone: Role of Glut-1 Activation. Biochim. Biophys. Acta.

[52] Nellis M.M., Doering C.B., Kasinski A., Danner D.J. (2002). Insulin Increases Branched-Chain Alpha-Ketoacid Dehydrogenase Kinase Expression in Clone 9 Rat Cells. Am. J. Physiol. Endocrinol. Metab..

[53] Gao M., Liu D. (2013). Resveratrol Suppresses T0901317-Induced Hepatic Fat Accumulation in Mice. AAPS J..

[54] Rauf A., Imran M., Butt M.S., Nadeem M., Peters D.G., Mubarak M.S. (2018). Resveratrol as an Anti-Cancer Agent: A Review. Crit. Rev. Food Sci. Nutr..

[55] Molinaro A., Becattini B., Solinas G. (2020). Insulin Signaling and Glucose Metabolism in Different Hepatoma Cell Lines Deviate from Hepatocyte Physiology toward a Convergent Aberrant Phenotype. Sci. Rep..

[56] Salmeen A., Andersen J.N., Myers M.P., Tonks N.K., Barford D. (2000). Molecular Basis for the Dephosphorylation of the Activation Segment of the Insulin Receptor by Protein Tyrosine Phosphatase 1b. Mol. Cell.

[57] Liao Y., Hung M.C. (2010). Physiological Regulation of Akt Activity and Stability. Am. J. Transl. Res..

[58] Chen X., Hu X., Li Y., Zhu C., Dong X., Zhang R., Ma J., Huang S., Chen L. (2019). Resveratrol Inhibits Erk1/2-Mediated Adhesion of Cancer Cells Via Activating Pp2a–Pten Signaling Network. J. Cell. Physiol..

[59] Venkatesan B., Ghosh-Choudhury N., Das F., Mahimainathan L., Kamat A., Kasinath B.S., Abboud H.E., Choudhury G.G. (2008). Resveratrol Inhibits Pdgf Receptor Mitogenic Signaling in Mesangial Cells: Role of Ptp1b. FASEB J..

[60] Ye M.-J., Meng N. (2021). Resveratrol Acts Via the Mitogen-Activated Protein Kinase (Mapk) Pathway to Protect Retinal Ganglion Cells from Apoptosis Induced by Hydrogen Peroxide. Bioengineered.

[61] Liu C., Zhang R., Sun C., Zhang H., Xu C., Liu W., Gao W., Huang S., Chen L. (2015). Resveratrol Prevents Cadmium Activation of Erk1/2 and Jnk Pathways from Neuronal Cell Death Via Protein Phosphatases 2a and 5. J. Neurochem..

[62] Ko J.-H., Sethi G., Um J.-Y., Shanmugam M.K., Arfuso F., Kumar A.P., Bishayee A., Ahn K.S. (2017). The Role of Resveratrol in Cancer Therapy. Int. J. Mol. Sci..

[63] Wang Y., Romigh T., He X., Orloff M.S., Silverman R.H., Heston W.D., Eng C. (2010). Resveratrol Regulates the Pten/Akt Pathway through Androgen Receptor-Dependent and -Independent Mechanisms in Prostate Cancer Cell Lines. Hum. Mol. Genet..

[64] Jiang H., Shang X., Wu H., Gautam S.C., Al-Holou S., Li C., Kuo J., Zhang L., Chopp M. (2009). Resveratrol Downregulates Pi3k/Akt/Mtor Signaling Pathways in Human U251 Glioma Cells. J. Exp. Ther. Oncol..

[65] Lin H.Y., Shih A., Davis F.B., Tang H.Y., Martino L.J., Bennett J.A., Davis P.J. (2002). Resveratrol Induced Serine Phosphorylation of P53 Causes Apoptosis in a Mutant P53 Prostate Cancer Cell Line. J. Urol..

[66] Lin C., Crawford D.R., Lin S., Hwang J., Sebuyira A., Meng R., Westfall J.E., Tang H.-Y., Lin S., Yu P.-Y. (2011). Inducible Cox-2-Dependent Apoptosis in Human Ovarian Cancer Cells. Carcinogenesis.

[67] Zhang S., Cao H.J., Davis F.B., Tang H.-Y., Davis P.J., Lin H.-Y. (2004). Oestrogen Inhibits Resveratrol-Induced Post-Translational Modification of P53 and Apoptosis in Breast Cancer Cells. Br. J. Cancer.

[68] Shimizu T., Nakazato T., Xian M.J., Sagawa M., Ikeda Y., Kizaki M. (2006). Resveratrol Induces Apoptosis of Human Malignant B Cells by Activation of Caspase-3 and P38 Map Kinase Pathways. Biochem. Pharmacol..

[69] Miloso M., Bertelli A.A.E., Nicolini G., Tredici G. (1999). Resveratrol-Induced Activation of the Mitogen-Activated Protein Kinases, Erk1 and Erk2, in Human Neuroblastoma Sh-Sy5y Cells. Neurosci. Lett..

[70] Johnson H., Narayan S., Sharma A.K. (2024). Altering Phosphorylation in Cancer through Pp2a Modifiers. Cancer Cell Int..

[71] Mazhar S., Taylor S.E., Sangodkar J., Narla G. (2019). Targeting Pp2a in Cancer: Combination Therapies. Biochim. Biophys. Acta Mol. Cell Res..

[72] Zeng Y.-H., Zhou L.-Y., Chen Q.-Z., Li Y., Shao Y., Ren W.-Y., Liao Y.-P., Wang H., Zhu J.-H., Huang M. (2017). Resveratrol Inactivates Pi3k/Akt Signaling through Upregulating Bmp7 in Human Colon Cancer Cells. Oncol. Rep..

[73] Dirimanov S., Högger P. (2019). Screening of Inhibitory Effects of Polyphenols on Akt-Phosphorylation in Endothelial Cells and Determination of Structure-Activity Features. Biomolecules.

[74] Marr L., Biswas D., Daly L.A., Browning C., Vial S.C.M., Maskell D.P., Hudson C., Bertrand J.A., Pollard J., Ranson N.A. (2022). Mechanism of Glycogen Synthase Inactivation and Interaction with Glycogenin. Nat. Commun..

[75] Rozhkov S.V., Sharlo K.A., Shenkman B.S., Mirzoev T.M. (2022). The Role of Glycogen Synthase Kinase-3 in the Regulation of Ribosome Biogenesis in Rat Soleus Muscle under Disuse Conditions. Int. J. Mol. Sci..

[76] Gutierrez-Rodelo C., Roura-Guiberna A., Olivares-Reyes J.A. (2017). Molecular Mechanisms of Insulin Resistance: An Update. Gac. Med. Mex..

[77] Gutierrez-Rodelo C., Arellano-Plancarte A., Hernandez-Aranda J., Landa-Galvan H.V., Parra-Mercado G.K., Moreno-Licona N.J., Hernandez-Gonzalez K.D., Catt K.J., Villalobos-Molina R., Olivares-Reyes J.A. (2022). Angiotensin Ii Inhibits Insulin Receptor Signaling in Adipose Cells. Int. J. Mol. Sci..

[78] Li L., Sampat K., Hu N., Zakari J., Yuspa S.H. (2006). Protein Kinase C Negatively Regulates Akt Activity and Modifies Uvc-Induced Apoptosis in Mouse Keratinocytes. J. Biol. Chem..

[79] Samuel V.T., Liu Z.-X., Wang A., Beddow S.A., Geisler J.G., Kahn M., Zhang X.-M., Monia B.P., Bhanot S., Shulman G.I. (2007). Inhibition of Protein Kinase Cε Prevents Hepatic Insulin Resistance in Nonalcoholic Fatty Liver Disease. J. Clin. Investig..

[80] Aguirre V., Werner E.D., Giraud J., Lee Y.H., Shoelson S.E., White M.F. (2002). Phosphorylation of Ser307 in Insulin Receptor Substrate-1 Blocks Interactions with the Insulin Receptor and Inhibits Insulin Action. J. Biol. Chem..

[81] England K., Rumsby M.G. (2000). Changes in Protein Kinase C ∊ Phosphorylation Status and Intracellular Localization as 3t3 and 3t6 Fibroblasts Grow to Confluency and Quiescence: A Role for Phosphorylation at Ser-729?. Biochem. J..

[82] Zick Y. (2005). Ser/Thr Phosphorylation of Irs Proteins: A Molecular Basis for Insulin Resistance. Sci. Signal..

[83] Schmitz-Peiffer C., Biden T.J. (2008). Protein Kinase C Function in Muscle, Liver, and Beta-Cells and Its Therapeutic Implications for Type 2 Diabetes. Diabetes.

[84] Bossenmaier B., Mosthaf L., Mischak H., Ullrich A., Haring H.U. (1997). Protein Kinase C Isoforms β 1 and β 2 Inhibit the Tyrosine Kinase Activity of the Insulin Receptor. Diabetologia.

[85] Kawano T., Inokuchi J., Eto M., Murata M., Kang J.-H. (2021). Activators and Inhibitors of Protein Kinase C (Pkc): Their Applications in Clinical Trials. Pharmaceutics.

[86] Fang Z., Xu A., Wu L., Hei T.K., Hong M. (2016). The Role of Protein Kinase C Alpha Translocation in Radiation-Induced Bystander Effect. Sci. Rep..

[87] Sun X.J., Liu F. (2009). Phosphorylation of Irs Proteins Yin-Yang Regulation of Insulin Signaling. Vitam. Horm..

[88] Boura-Halfon S., Zick Y. (2009). Phosphorylation of Irs Proteins, Insulin Action, and Insulin Resistance. Am. J. Physiol. Endocrinol. Metab..

[89] Eleftheriou P., Geronikaki A., Petrou A. (2019). Ptp1b Inhibition, a Promising Approach for the Treatment of Diabetes Type Ii. Curr. Top. Med. Chem..

[90] Abdelsalam S.S., Korashy H.M., Zeidan A., Agouni A. (2019). The Role of Protein Tyrosine Phosphatase (Ptp)-1b in Cardiovascular Disease and Its Interplay with Insulin Resistance. Biomolecules.

[91] Ren L., Chen X., Luechapanichkul R., Selner N.G., Meyer T.M., Wavreille A.-S., Chan R., Iorio C., Zhou X., Neel B.G. (2011). Substrate Specificity of Protein Tyrosine Phosphatases 1b, Rptpα, Shp-1, and Shp-2. Biochemistry.

[92] Baur J.A., Pearson K.J., Price N.L., Jamieson H.A., Lerin C., Kalra A., Prabhu V.V., Allard J.S., Lopez-Lluch G., Lewis K. (2006). Resveratrol Improves Health and Survival of Mice on a High-Calorie Diet. Nature.

[93] Zhang Z., Lei B., Chai W., Liu R., Li T. (2019). Increased Expression of Insulin-Like Growth Factor-1 Receptor Predicts Poor Prognosis in Patients with Hepatocellular Carcinoma. Medicine.

[94] Cai W., Ma Y., Song L., Cao N., Gao J., Zhou S., Tang X. (2023). Igf-1r down Regulates the Sensitivity of Hepatocellular Carcinoma to Sorafenib through the Pi3k/Akt and RasRaf/Erk Signaling Pathways. BMC Cancer.

[95] Ngo M.-H.T., Jeng H.-Y., Kuo Y.-C., Diony Nanda J., Brahmadhi A., Ling T.-Y., Chang T.-S., Huang Y.-H. (2021). The Role of Igf/Igf-1r Signaling in Hepatocellular Carcinomas: Stemness-Related Properties and Drug Resistance. Int. J. Mol. Sci..

[96] Paraiso K.H., Van Der Kooi K., Messina J.L., Smalley K.S. (2010). Measurement of Constitutive Mapk and Pi3k/Akt Signaling Activity in Human Cancer Cell Lines. Methods Enzymol..

[97] Li H., Han G., Li X., Li B., Wu B., Jin H., Wu L., Wang W. (2021). Mapk-Rap1a Signaling Enriched in Hepatocellular Carcinoma Is Associated with Favorable Tumor-Infiltrating Immune Cells and Clinical Prognosis. Front. Oncol..

[98] Braicu C., Buse M., Busuioc C., Drula R., Gulei D., Raduly L., Rusu A., Irimie A., Atanasov A.G., Slaby O. (2019). A Comprehensive Review on Mapk: A Promising Therapeutic Target in Cancer. Cancers.

[99] Van Horn D.J., Myers M.G., Backer J.M. (1994). Direct Activation of the Phosphatidylinositol 3′-Kinase by the Insulin Receptor. J. Biol. Chem..

[100] Staubs P.A., Reichart D.R., Saltiel A.R., Milarski K.L., Maegawa H., Berhanu P., Olefsky J.M., Seely B.L. (1994). Localization of the Insulin Receptor Binding Sites for the Sh2 Domain Proteins P85, Syp, and Gap. J. Biol. Chem..

[101] Yamamoto-Honda R., Honda Z.I., Ueki K., Tobe K., Kaburagi Y., Takahashi Y., Tamemoto H., Suzuki T., Itoh K., Akanuma Y. (1996). Mutant of Insulin Receptor Substrate-1 Incapable of Activating Phosphatidylinositol 3-Kinase Did Not Mediate Insulin-Stimulated Maturation of Xenopus Laevis Oocytes. J. Biol. Chem..

[102] Tartare-Deckert S., Murdaca J., Sawka-Verhelle D., Holt K.H., Pessin J.E., Obberghen E.V. (1996). Interaction of the Molecular Weight 85k Regulatory Subunit of the Phosphatidylinositol 3-Kinase with the Insulin Receptor and the Insulin-like Growth Factor-1 (Igf- I) Receptor: Comparative Study Using the Yeast Two-Hybrid System. Endocrinology.

[103] Yuan T.L., Cantley L.C. (2008). Pi3k Pathway Alterations in Cancer: Variations on a Theme. Oncogene.

[104] Liu R., Chen Y., Liu G., Li C., Song Y., Cao Z., Li W., Hu J., Lu C., Liu Y. (2020). Pi3k/Akt Pathway as a Key Link Modulates the Multidrug Resistance of Cancers. Cell Death Dis..

[105] Tian L.-Y., Smit D.J., Jücker M. (2023). The Role of Pi3k/Akt/Mtor Signaling in Hepatocellular Carcinoma Metabolism. Int. J. Mol. Sci..

[106] Sun E.J., Wankell M., Palamuthusingam P., McFarlane C., Hebbard L. (2021). Targeting the Pi3k/Akt/Mtor Pathway in Hepatocellular Carcinoma. Biomedicines.

[107] Chetram M.A., Hinton C.V. (2012). Pten Regulation of Erk1/2 Signaling in Cancer. J. Recept. Signal Transduct..

[108] Kuo Y.-C., Huang K.-Y., Yang C.-H., Yang Y.-S., Lee W.-Y., Chiang C.-W. (2008). Regulation of Phosphorylation of Thr-308 of Akt, Cell Proliferation, and Survival by the B55α Regulatory Subunit Targeting of the Protein Phosphatase 2a Holoenzyme to Akt. J. Biol. Chem..

[109] Resjö S., Göransson O., Härndahl L., Zolnierowicz S., Manganiello V., Degerman E. (2002). Protein Phosphatase 2a Is the Main Phosphatase Involved in the Regulation of Protein Kinase B in Rat Adipocytes. Cell. Signal..

[110] Schweiger S., Matthes F., Posey K., Kickstein E., Weber S., Hettich M.M., Pfurtscheller S., Ehninger D., Schneider R., Krauß S. (2017). Resveratrol Induces Dephosphorylation of Tau by Interfering with the Mid1-Pp2a Complex. Sci. Rep..

[111] Jian B., Yang S., Chaudry I.H., Raju R. (2014). Resveratrol Restores Sirtuin 1 (Sirt1) Activity and Pyruvate Dehydrogenase Kinase 1 (Pdk1) Expression after Hemorrhagic Injury in a Rat Model. Mol. Med..

[112] Alayev A., Doubleday P.F., Berger S.M., Ballif B.A., Holz M.K. (2014). Phosphoproteomics Reveals Resveratrol-Dependent Inhibition of Akt/Mtorc1/S6k1 Signaling. J. Proteome Res..

[113] Miao R., Fang X., Wei J., Wu H., Wang X., Tian J. (2022). Akt: A Potential Drug Target for Metabolic Syndrome. Front. Physiol..

[114] Rayasam G.V., Tulasi V.K., Sodhi R., Davis J.A., Ray A. (2009). Glycogen Synthase Kinase 3: More Than a Namesake. Br. J. Pharmacol..

[115] Wang L., Li J., Di L.J. (2022). Glycogen Synthesis and Beyond, a Comprehensive Review of Gsk3 as a Key Regulator of Metabolic Pathways and a Therapeutic Target for Treating Metabolic Diseases. Med. Res. Rev..

[116] Papadopoli D., Pollak M., Topisirovic I. (2021). The Role of Gsk3 in Metabolic Pathway Perturbations in Cancer. Biochim. Biophys. Acta Mol. Cell Res..

[117] Zhang H., Wang W., Fang H., Yang Y., Li X., He J., Jiang X., Wang W., Liu S., Hu J. (2014). Gsk-3β Inhibition Attenuates Clp-Induced Liver Injury by Reducing Inflammation and Hepatic Cell Apoptosis. Mediat. Inflamm..

[118] Irimia J.M., Meyer C.M., Segvich D.M., Surendran S., DePaoli-Roach A.A., Morral N., Roach P.J. (2017). Lack of Liver Glycogen Causes Hepatic Insulin Resistance and Steatosis in Mice. J. Biol. Chem..

[119] Patel S., Macaulay K., Woodgett J.R. (2011). Tissue-Specific Analysis of Glycogen Synthase Kinase-3α (Gsk-3α) in Glucose Metabolism: Effect of Strain Variation. PLoS ONE.

[120] Ciaraldi T.P., Nikoulina S.E., Bandukwala R.A., Carter L., Henry R.R. (2007). Role of Glycogen Synthase Kinase-3α in Insulin Action in Cultured Human Skeletal Muscle Cells. Endocrinology.

[121] Cortés-Vieyra R., Silva-García O., Gómez-García A., Gutiérrez-Castellanos S., Álvarez-Aguilar C., Baizabal-Aguirre V.M. (2021). Glycogen Synthase Kinase 3β Modulates the Inflammatory Response Activated by Bacteria, Viruses, and Parasites. Front. Immunol..

[122] Zhang D., Shi C., Zhang Q., Wang Y., Guo J., Gong Z. (2023). Inhibition of Gsk3β Activity Alleviates Acute Liver Failure Via Suppressing Multiple Programmed Cell Death. J. Inflamm..

[123] Ren F., Zhang L., Zhang X., Shi H., Wen T., Bai L., Zheng S., Chen Y., Chen D., Li L. (2016). Inhibition of Glycogen Synthase Kinase 3β Promotes Autophagy to Protect Mice from Acute Liver Failure Mediated by Peroxisome Proliferator-Activated Receptor α. Cell Death Dis..

[124] Ludvik B., Nolan J.J., Roberts A., Baloga J., Joyce M., Bell J.M., Olefsky J.M. (1995). A Noninvasive Method to Measure Splanchnic Glucose Uptake after Oral Glucose Administration. J. Clin. Investig..

[125] Goldstein B.J., Bittner-Kowalczyk A., White M.F., Harbeck M. (2000). Tyrosine Dephosphorylation and Deactivation of Insulin Receptor Substrate-1 by Protein-Tyrosine Phosphatase 1b. J. Biol. Chem..

[126] Steinberg S.F. (2015). Mechanisms for Redox-Regulation of Protein Kinase C. Front. Pharmacol..

[127] Steinberg S.F. (2008). Structural Basis of Protein Kinase C Isoform Function. Physiol. Rev..

[128] Ueda Y., Hirai S.-I., Osada S.-I., Suzuki A., Mizuno K., Ohno S. (1996). Protein Kinase C δ Activates the Mek-Erk Pathway in a Manner Independent of Ras and Dependent on Raf. J. Biol. Chem..

[129] Letiges M., Plomann M., Standaert M.L., Bandyopadhyay G., Sajan M.P., Kanoh Y., Farese R.V. (2002). Knockout of Pkcα Enhances Insulin Signaling through Pi3k. Mol. Endocrinol..

[130] Maeno Y., Li Q., Park K., Rask-Madsen C., Gao B., Matsumoto M., Liu Y., Wu I.H., White M.F., Feener E.P. (2012). Inhibition of Insulin Signaling in Endothelial Cells by Protein Kinase C-Induced Phosphorylation of P85 Subunit of Phosphatidylinositol 3-Kinase (Pi3k). J. Biol. Chem..

[131] Turban S., Hajduch E. (2011). Protein Kinase C Isoforms: Mediators of Reactive Lipid Metabolites in the Development of Insulin Resistance. FEBS Lett..

[132] Considine R.V., Nyce M.R., Allen L.E., Morales L.M., Triester S., Serrano J., Colberg J., Lanza-Jacoby S., Caro J.F. (1995). Protein Kinase C Is Increased in the Liver of Humans and Rats with Non-Insulin-Dependent Diabetes Mellitus: An Alteration Not Due to Hyperglycemia. J. Clin. Investig..

[133] Wen-Sheng W., Jun-Ming H. (2005). Activation of Protein Kinase C Alpha Is Required for Tpa-Triggered Erk (Mapk) Signaling and Growth Inhibition of Human Hepatoma Cell Hepg2. J. Biomed. Sci..

[134] Wu W. (2006). Protein Kinase C α Trigger Ras and Raf-Independent Mek/Erk Activation for Tpa-Induced Growth Inhibition of Human Hepatoma Cell Hepg2. Cancer Lett..

[135] Rucci N., Digiacinto C., Orrù L., Millimaggi D., Baron R., Teti A. (2005). A Novel Protein Kinase C α-Dependent Signal to Erk1/2 Activated by αvβ3 Integrin in Osteoclasts and in Chinese Hamster Ovary (Cho) Cells. J. Cell Sci..

[136] Cheng J.-J., Wung B.-S., Chao Y.-J., Wang D.L. (2001). Sequential Activation of Protein Kinase C (Pkc)-α and Pkc-ε Contributes to Sustained Raf/Erk1/2 Activation in Endothelial Cells under Mechanical Strain. J. Biol. Chem..

[137] Guo K., Liu Y., Zhou H., Dai Z., Zhang J., Sun R., Chen J., Sun Q., Lu W., Kang X. (2008). Involvement of Protein Kinase C β–Extracellular Signal-Regulating Kinase_1/2_P38 Mitogen-Activated Protein Kinase–Heat Shock Protein 27 Activation in Hepatocellular Carcinoma Cell Motility and Invasion. Cancer Sci..

[138] Trollér U., Zeidman R., Svensson K., Larsson C. (2001). A Pkcβ Isoform Mediates Phorbol Ester-Induced Activation of Erk1/2 and Expression of Neuronal Differentiation Genes in Neuroblastoma Cells. FEBS Lett..

[139] Berti L., Mosthaf L., Kroder G., Kellerer M., Tippmer S., Mushack J., Seffer E., Seedorf K., Haring H. (1994). Glucose-Induced Translocation of Protein Kinase C Isoforms in Rat-1 Fibroblasts Is Paralleled by Inhibition of the Insulin Receptor Tyrosine Kinase. J. Biol. Chem..

[140] Kellerer M., Mushack J., Seffer E., Mischak H., Ullrich A., Haring H.U. (1998). Protein Kinase C Isoforms Alpha, Delta and Theta Require Insulin Receptor Substrate-1 to Inhibit the Tyrosine Kinase Activity of the Insulin Receptor in Human Kidney Embryonic Cells (Hek 293 Cells). Diabetologia.

[141] Cipok M., Aga-Mizrachi S., Bak A., Feurstein T., Steinhart R., Brodie C., Sampson S.R. (2006). Protein Kinase Cα Regulates Insulin Receptor Signaling in Skeletal Muscle. Biochem. Biophys. Res. Commun..

[142] Bakke J., Haj F.G. (2015). Protein-Tyrosine Phosphatase 1b Substrates and Metabolic Regulation. Semin. Cell Dev. Biol..

[143] Haj F.G., Zabolotny J.M., Kim Y.-B., Kahn B.B., Neel B.G. (2005). Liver-Specific Protein-Tyrosine Phosphatase 1b (Ptp1b) Re-Expression Alters Glucose Homeostasis of Ptp1b-/-Mice. J. Biol. Chem..

[144] Zinker B.A., Rondinone C.M., Trevillyan J.M., Gum R.J., Clampit J.E., Waring J.F., Xie N., Wilcox D., Jacobson P., Frost L. (2002). Ptp1b Antisense Oligonucleotide Lowers Ptp1b Protein, Normalizes Blood Glucose, and Improves Insulin Sensitivity in Diabetic Mice. Proc. Natl. Acad. Sci. USA.

[145] González-Rodríguez Á., Gutierrez J.A.M., Sanz-González S., Ros M., Burks D.J., Valverde Á.M. (2010). Inhibition of Ptp1b Restores Irs1-Mediated Hepatic Insulin Signaling in Irs2-Deficient Mice. Diabetes.

[146] Dadke S., Kusari J., Chernoff J. (2000). Down-Regulation of Insulin Signaling by Protein-Tyrosine Phosphatase 1b Is Mediated by an N-Terminal Binding Region. J. Biol. Chem..

[147] Liu R., Mathieu C., Berthelet J., Zhang W., Dupret J.-M., Rodrigues Lima F. (2022). Human Protein Tyrosine Phosphatase 1b (Ptp1b): From Structure to Clinical Inhibitor Perspectives. Int. J. Mol. Sci..

[148] Bheri M., Pandey G.K. (2019). Protein Phosphatases Meet Reactive Oxygen Species in Plant Signaling Networks. Environ. Exp. Bot..

[149] Corcoran A., Cotter T.G. (2013). Redox Regulation of Protein Kinases. FEBS J..

[150] Lou Y.W., Chen Y.Y., Hsu S.F., Chen R.K., Lee C.L., Khoo K.H., Tonks N.K., Meng T.C. (2008). Redox Regulation of the Protein Tyrosine Phosphatase Ptp1b in Cancer Cells. FEBS J..

[151] Giorgi C., Agnoletto C., Baldini C., Bononi A., Bonora M., Marchi S., Missiroli S., Patergnani S., Poletti F., Rimessi A. (2010). Redox Control of Protein Kinase C: Cell- and Disease-Specific Aspects. Antioxid. Redox Signal..

[152] Truong T.H., Carroll K.S. (2013). Redox Regulation of Protein Kinases. Crit. Rev. Biochem. Mol. Biol..

[153] Finkel T. (2011). Signal Transduction by Reactive Oxygen Species. J. Cell Biol..

[154] Haque A., Andersen J.N., Salmeen A., Barford D., Tonks N.K. (2011). Conformation-Sensing Antibodies Stabilize the Oxidized Form of Ptp1b and Inhibit Its Phosphatase Activity. Cell.

[155] Krishnan N., Bonham C.A., Rus I.A., Shrestha O.K., Gauss C.M., Haque A., Tocilj A., Joshua-Tor L., Tonks N.K. (2018). Harnessing Insulin- and Leptin-Induced Oxidation of Ptp1b for Therapeutic Development. Nat. Commun..

[156] Londhe A.D., Bergeron A., Curley S.M., Zhang F., Rivera K.D., Kannan A., Coulis G., Rizvi S.H.M., Kim S.J., Pappin D.J. (2020). Regulation of Ptp1b Activation through Disruption of Redox-Complex Formation. Nat. Chem. Biol..

[157] Senga T., Miyazaki K., Machida K., Iwata H., Matsuda S., Nakashima I., Hamaguchi M. (2000). Clustered Cysteine Residues in the Kinase Domain of V-Src: Critical Role for Protein Stability, Cell Transformation and Sensitivity to Herbimycin A. Oncogene.

[158] Giannoni E., Buricchi F., Raugei G., Ramponi G., Chiarugi P. (2005). Intracellular Reactive Oxygen Species Activate Src Tyrosine Kinase during Cell Adhesion and Anchorage-Dependent Cell Growth. Mol. Cell. Biol..

[159] Giannoni E., Taddei M.L., Chiarugi P. (2010). Src Redox Regulation: Again in the Front Line. Free Radic. Biol. Med..

[160] Wani R., Bharathi N.S., Field J., Tsang A.W., Furdui C.M. (2011). Oxidation of Akt2 Kinase Promotes Cell Migration and Regulates G1-S Transition in the Cell Cycle. Cell Cycle.

[161] Wani R., Qian J., Yin L., Bechtold E., King S.B., Poole L.B., Paek E., Tsang A.W., Furdui C.M. (2011). Isoform-Specific Regulation of Akt by Pdgf-Induced Reactive Oxygen Species. Proc. Natl. Acad. Sci. USA.

[162] Murata H., Ihara Y., Nakamura H., Yodoi J., Sumikawa K., Kondo T. (2003). Glutaredoxin Exerts an Antiapoptotic Effect by Regulating the Redox State of Akt. J. Biol. Chem..

[163] Galli S., Antico Arciuch V.G., Poderoso C., Converso D.P., Zhou Q., De Kier Joffé E.B., Cadenas E., Boczkowski J., Carreras M.C., Poderoso J.J. (2008). Tumor Cell Phenotype Is Sustained by Selective Mapk Oxidation in Mitochondria. PLoS ONE.

[164] Paulsen C.E., Truong T.H., Garcia F.J., Homann A., Gupta V., Leonard S.E., Carroll K.S. (2012). Peroxide-Dependent Sulfenylation of the Egfr Catalytic Site Enhances Kinase Activity. Nat. Chem. Biol..

[165] Truong T.H., Carroll K.S. (2012). Redox Regulation of Epidermal Growth Factor Receptor Signaling through Cysteine Oxidation. Biochemistry.

[166] Cosentino-Gomes D., Rocco-Machado N., Meyer-Fernandes J.R. (2012). Cell Signaling through Protein Kinase C Oxidation and Activation. Int. J. Mol. Sci..

[167] Martelli A.M., Evangelisti C., Nyakern M., Manzoli F.A. (2006). Nuclear Protein Kinase C. Biochim. Biophys. Acta Mol. Cell Biol. Lipids.

[168] Hsu A.H., Lum M.A., Shim K.-S., Frederick P.J., Morrison C.D., Chen B., Lele S.M., Sheinin Y.M., Daikoku T., Dey S.K. (2018). Crosstalk between Pkcα and Pi3k/Akt Signaling Is Tumor Suppressive in the Endometrium. Cell Rep..

[169] Nadel G., Yao Z., Hacohen-Lev-Ran A., Wainstein E., Maik-Rachline G., Ziv T., Naor Z., Admon A., Seger R. (2024). Phosphorylation of Pp2ac by Pkc Is a Key Regulatory Step in the Pp2a-Switch-Dependent Akt Dephosphorylation That Leads to Apoptosis. Cell Commun. Signal..

[170] Hevener A.L., Ribas V., Moore T.M., Zhou Z. (2021). Erα in the Control of Mitochondrial Function and Metabolic Health. Trends Mol. Med..

[171] González C., Alonso A., Fernández R., Patterson A.M. (2003). Regulation of Insulin Receptor Substrate-1 in the Liver, Skeletal Muscle and Adipose Tissue of Rats Throughout Pregnancy. Gynecol. Endocrinol..

[172] Tran H.T., Liong S., Lim R., Barker G., Lappas M. (2017). Resveratrol Ameliorates the Chemical and Microbial Induction of Inflammation and Insulin Resistance in Human Placenta, Adipose Tissue and Skeletal Muscle. PLoS ONE.

[173] Bo T., Gao L., Yao Z., Shao S., Wang X., Proud C.G., Zhao J. (2024). Hepatic Selective Insulin Resistance at the Intersection of Insulin Signaling and Metabolic Dysfunction-Associated Steatotic Liver Disease. Cell Metab..

[174] Michael M.D., Kulkarni R.N., Postic C., Previs S.F., Shulman G.I., Magnuson M.A., Kahn C.R. (2000). Loss of Insulin Signaling in Hepatocytes Leads to Severe Insulin Resistance and Progressive Hepatic Dysfunction. Mol. Cell.

[175] Brown M.S., Goldstein J.L. (2008). Selective Versus Total Insulin Resistance: A Pathogenic Paradox. Cell Metab..

[176] Li S., Brown M.S., Goldstein J.L. (2010). Bifurcation of Insulin Signaling Pathway in Rat Liver: Mtorc1 Required for Stimulation of Lipogenesis, but Not Inhibition of Gluconeogenesis. Proc. Natl. Acad. Sci. USA.

[177] Titchenell P.M., Quinn W.J., Lu M., Chu Q., Lu W., Li C., Chen H., Monks B.R., Chen J., Rabinowitz J.D. (2016). Direct Hepatocyte Insulin Signaling Is Required for Lipogenesis but Is Dispensable for the Suppression of Glucose Production. Cell Metab..

[178] Li Z., Tian Z., Shi X., Long A., Wang Y., Yang Y., Wang Y., Zhang J., Wang Y. (2025). Adipose Tissue-Derived Prxl2a Suppresses Hepatic Lipogenesis in a Study with Male Mice. Nat. Commun..

[179] Duan Y., Yang Y., Zhao S., Bai Y., Yao W., Gao X., Yin J. (2024). Crosstalk in Extrahepatic and Hepatic System in Nafld/Nash. Liver Int..

[180] Song Z., Xiaoli A.M., Yang F. (2018). Regulation and Metabolic Significance of De Novo Lipogenesis in Adipose Tissues. Nutrients.

[181] Pandit H., Li Y., Li X., Zhang W., Li S., Martin R.C.G. (2018). Enrichment of Cancer Stem Cells Via β-Catenin Contributing to the Tumorigenesis of Hepatocellular Carcinoma. BMC Cancer.

[182] Landa-Galvan H.V., Rios-Castro E., Romero-Garcia T., Rueda A., Olivares-Reyes J.A. (2020). Metabolic Syndrome Diminishes Insulin-Induced Akt Activation and Causes a Redistribution of Akt-Interacting Proteins in Cardiomyocytes. PLoS ONE.

[183] Horinouchi T., Hoshi A., Harada T., Higa T., Karki S., Terada K., Higashi T., Mai Y., Nepal P., Mazaki Y. (2016). Endothelin-1 Suppresses Insulin-Stimulated Akt Phosphorylation and Glucose Uptake Via Gpcr Kinase 2 in Skeletal Muscle Cells. Br. J. Pharmacol..

[184] Sun Z.J., Pan C.E., Liu H.S., Wang G.J. (2002). Anti-Hepatoma Activity of Resveratrol in Vitro. World J. Gastroenterol..

[185] Kasprzak-Drozd K., Niziński P., Kasprzak P., Kondracka A., Oniszczuk T., Rusinek A., Oniszczuk A. (2024). Does Resveratrol Improve Metabolic Dysfunction-Associated Steatotic Liver Disease (Masld)?. Int. J. Mol. Sci..

[186] Tomé-Carneiro J., Larrosa M., González-Sarrías A., Tomás-Barberán F., García-Conesa M., Espín J. (2013). Resveratrol and Clinical Trials: The Crossroad from in Vitro Studies to Human Evidence. Curr. Pharm. Des..

[187] Robinson K., Mock C., Liang D. (2015). Pre-Formulation Studies of Resveratrol. Drug Dev. Ind. Pharm..

[188] Szkudelska K., Szkudelski T. (2010). Resveratrol, Obesity and Diabetes. Eur. J. Pharmacol..

[189] Parra-Mercado G.K., Fuentes-Gonzalez A.M., Hernandez-Aranda J., Diaz-Coranguez M., Dautzenberg F.M., Catt K.J., Hauger R.L., Olivares-Reyes J.A. (2019). Crf1 Receptor Signaling Via the Erk1/2-Map and Akt Kinase Cascades: Roles of Src, Egf Receptor, and Pi3-Kinase Mechanisms. Front. Endocrinol..

