# Supplementary material for: Resveratrol Impairs Insulin Signaling in Hepatic Cells via Activation of PKC and PTP1B Pathways
Source: Int J Mol Sci. 2025 Aug 1;26(15):7434. doi: 10.3390/ijms26157434 (PMC12347301; doi:10.3390/ijms26157434)
Supplement: Supplementary file 1 [file ijms-26-07434-s001.zip › ijms-3706637-supplementary.pdf]

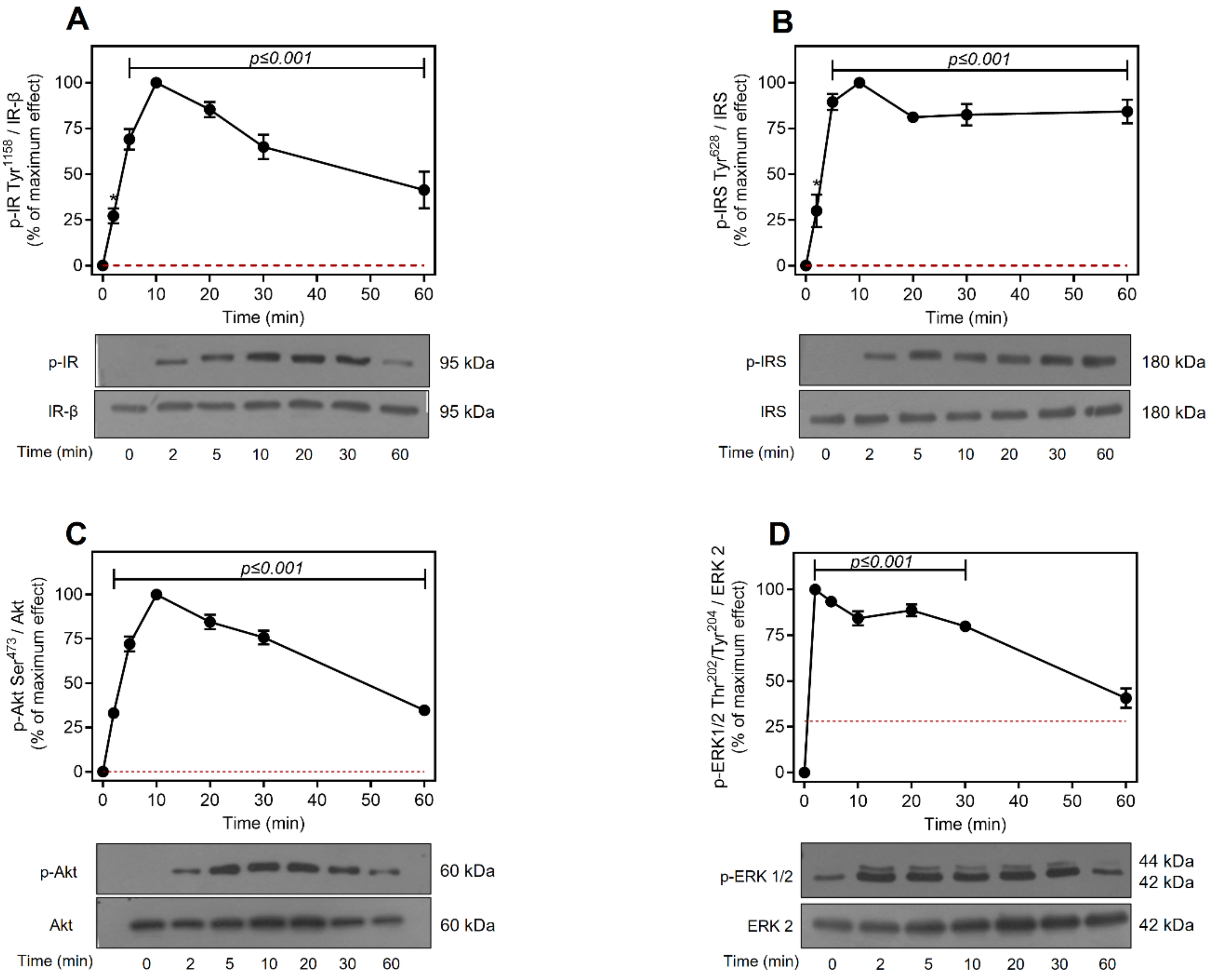

Supplementary Figure S1. Activation of the insulin pathway in Hepa 1-6. Cells were stimulated with 100 nM insulin for the indicated time (0-60 min). Total cell lysates were separated by SDS-PAGE and analyzed by immunoblotting using anti-p-IR-Tyr^{1158} **(A),** anti-p-IRS-Tyr^{628} **(B),** anti-p-Akt-Ser^{473} **(C),** and anti-p-ERK1/2-Thr^{202}/Tyr^{204} **(D)**, as described in Materials and Methods. Western blots were also probed for total IR, IRS, Akt, and ERK1/2 showing equal loading. Data represent the mean ± SEM of five individual experiments, and the panels below show representative immunoblots. Vertical lines represent SEM values. The *p-value* ≤0.001 indicates Ins vs. basal phosphorylation at 0 min (red dotted line).

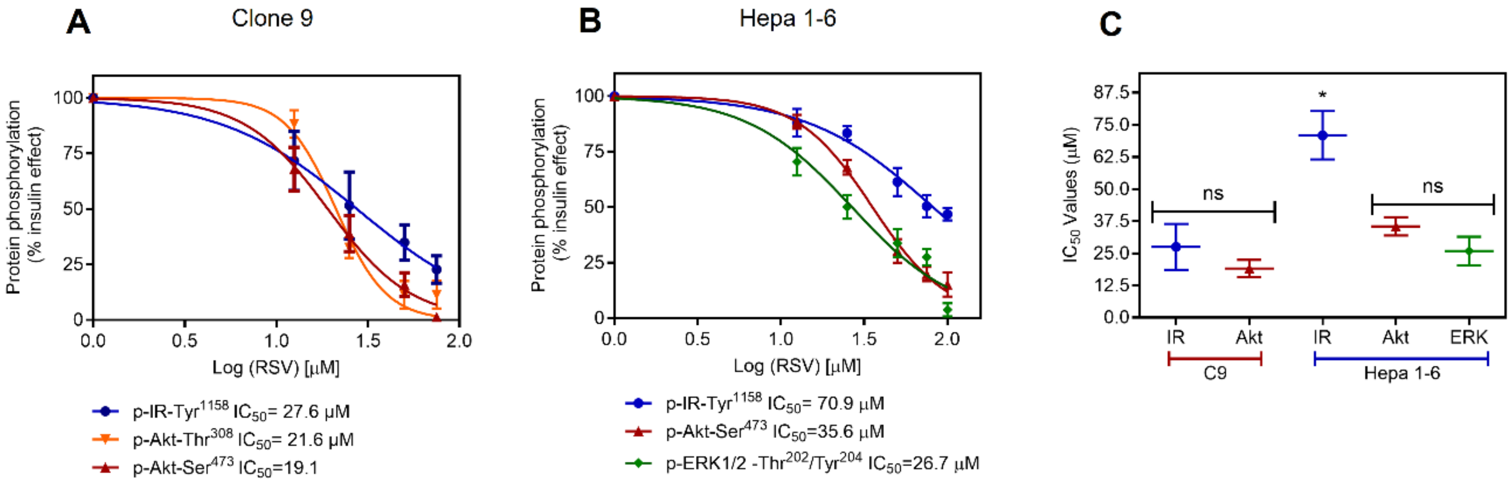

Supplementary Figure S2. Inhibitory concentration curves of RSV on the phosphorylation of insulin pathway proteins. Values of the RSV concentrations used (12.5, 25, 50, 75, and 100 µM) were transformed to their logarithms, as indicated, and the phosphorylation values of each protein were normalized as response values of 0-100%. Insulin-induced phosphorylation was considered 100% of the response. Insulin-induced phosphorylation in Hepa 1-6 **(A)** and C9 cells **(B). (C)**. The IC_{50} values of RSV were compared for phosphorylation of IR, IRS, Akt, and ERK 1/2. Data represent the mean ± SEM of five to six individual experiments. Vertical lines represent SEM values. *p<0.005 vs. Akt/ERK. ^{ns} represents no significant differences.

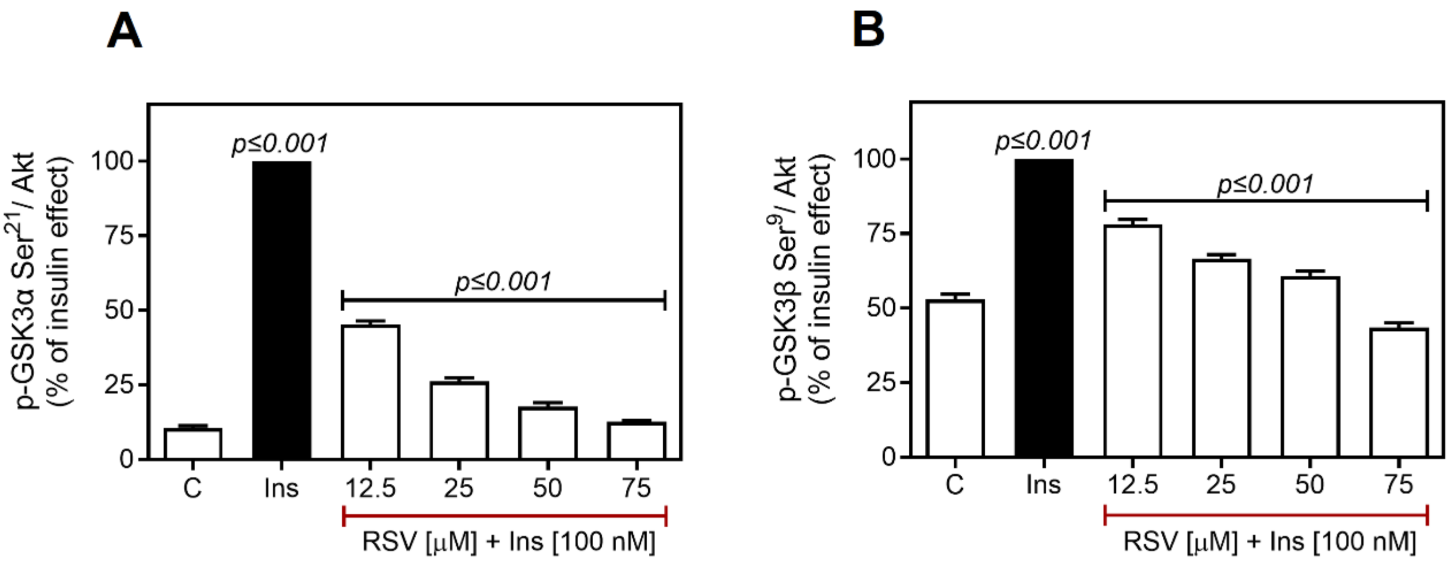

Supplementary Figure S3. Role of PKC in the RSV-mediated regulation of GSK3β/α. C9 cells were pretreated with or without BIM for 30 min, and then treated with different concentrations of RSV (as indicated) for 30 min. Finally, the cells were stimulated with 100 nM insulin for an additional 10 min. Under all experimental conditions, untreated cells were used as controls. Total cell lysates were separated by SD-PAGE and analyzed by immunoblotting with anti-p-GSK3β/α-Ser$^{21/9}$, as described in Materials and Methods. Western blots were also probed for total Akt showing equal loading. The bands corresponding to GSK3α-Ser^{21} and GSK3β-Ser^{9}, shown in Figure 5D, were analyzed separately. Data represent the mean ± SEM of five to six individual experiments. The *p-value* indicates Ins vs. C (basal phosphorylation) (**A, B**); RSV [12.5–75 µM] + Ins [100 nM] vs. Ins (**A, B**).

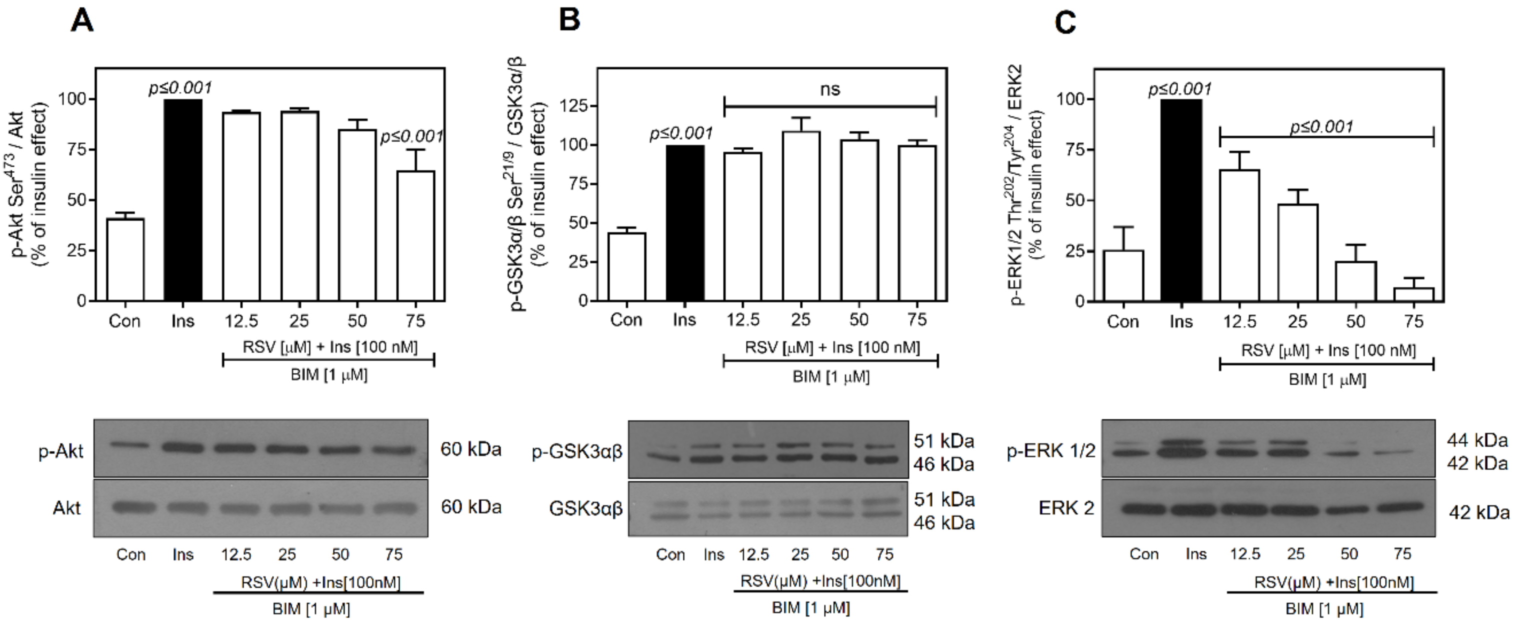

Supplementary Figure S4. Inhibition of PKC by BIM prevents the effect of RSV. C9 cells were pretreated with or without BIM for 30 min, and then treated with different concentrations of RSV (as indicated) for 30 min. Finally, the cells were stimulated with 100 nM insulin for an additional 10 min. Under all experimental conditions, untreated cells were used as controls. Total cell lysates were separated by SD-PAGE and analyzed by immunoblotting with anti-p-Akt-Ser^{473} **(A)**, anti-p-GSK3β/α-Ser$^{21/9}$ **(B)**, or anti-p-ERK1/2-Thr^{202}/Tyr^{204} **(C)**, as described in Materials and Methods. Western blots were also probed for total Akt, GSK3β/α, and ERK2 showing equal loading. Data represent the mean ± SEM of five to six individual experiments, and the panels below show representative immunoblots. The *p-value* indicates Ins vs. Con (basal phosphorylation) (**A-C**); BIM + RSV [12.5–75 μM] + Ins vs. Ins (**A-C**).

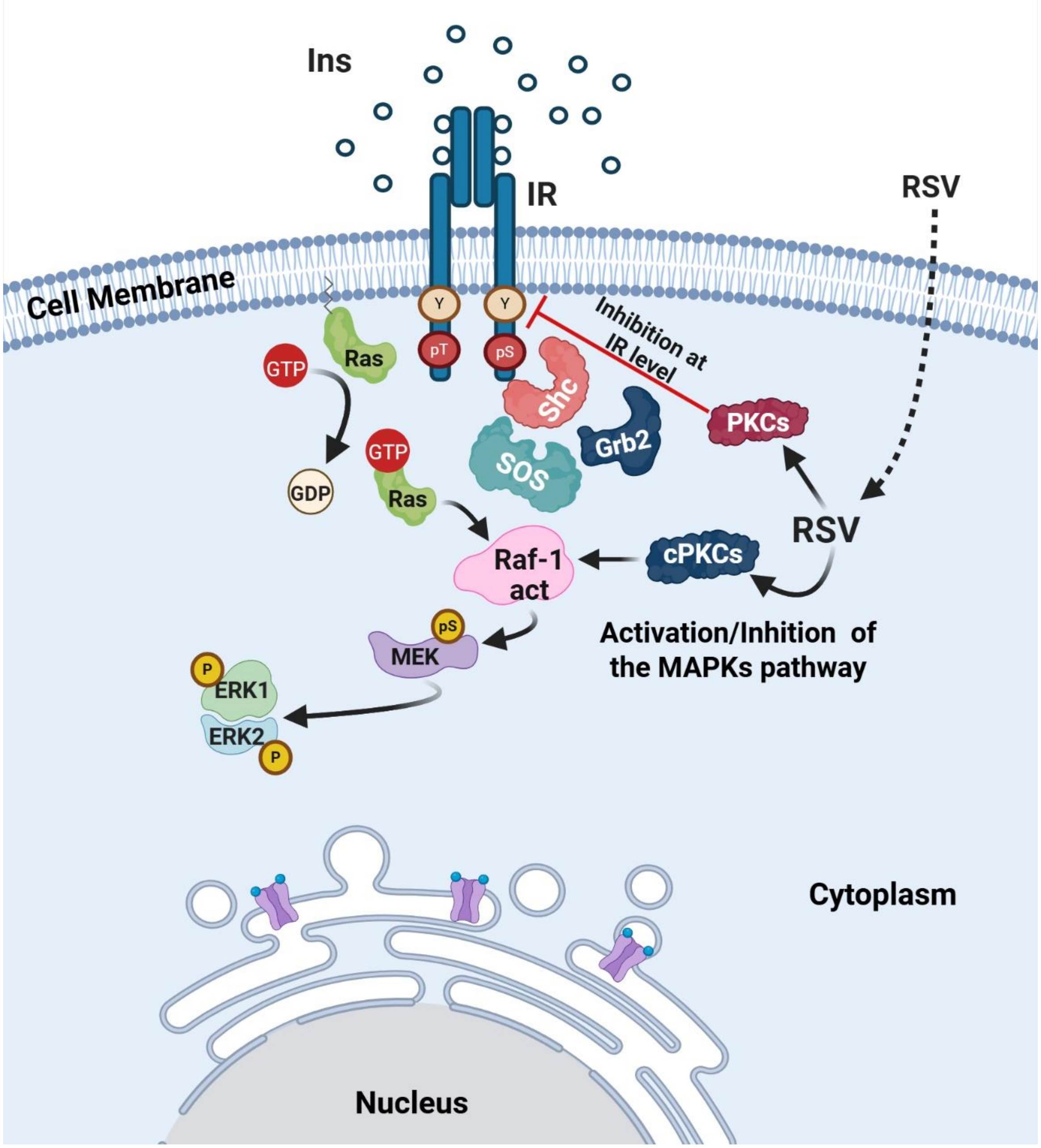

Supplementary Figure S5. Model of MAPK pathway activation by RSV in C9 cells. When hepatic C9 cells are treated with RSV, classical PKC isoforms are activated, which inhibits the PI3K/Akt pathway. RSV can also activate classical PKC (cPKC), which regulates the activation of ERK1/2 through Raf-1 and MEK. Black arrows indicate normal activation of MAPKs by insulin, and green arrows indicate the direct positive regulation of Raf-1 and MEK by PKC activated by RSV. Ins, insulin; nPKC, classical PKC; nPKCs, novel PKCs; pSer, serine phosphorylation; pThr, threonine phosphorylation; pTyr, tyrosine phosphorylation; Raf-1 act, Raf-1 activated; RSV, resveratrol.

Supplementary Table S1. Table of antibodies for Western blot and *immunoprecipitation studies.

Primary antibodies	*Source*	*Catalog no.*	*Clone*	*Dilutions*
p-Erk 1/2 (Thr^{202}/Tyr^{204})	Cell Signaling	9106L	Mouse monoclonal	1:15000
p-GS (Ser^{641})	Cell Signaling	3891	Rabbit polyclonal	1:5000
p-GSK3 α/β ($Ser^{21/9}$)	Cell Signaling	9331	Rabbit polyclonal	1:6000
p-Ser-PKC Substrate*	Cell Signaling	2261	Rabbit monoclonal	1:2000
p-Akt (Ser^{473})	Santa Cruz	sc-7985	Rabbit polyclonal	1:8000
p-Akt (Thr^{308})	Santa Cruz	sc-135650	Rabbit polyclonal	1:10000
p-IRS-1 (Tyr^{632})	Santa Cruz	sc-17196	Rabbit polyclonal	1:3000
Akt1/2/3	Santa Cruz	sc-8312	Rabbit polyclonal	1:10000
IRβ*	Santa Cruz	sc-711	Rabbit polyclonal	1:5000
IRS-1	Santa Cruz	sc-559	Rabbit polyclonal	1:5000
ERK 2	Santa Cruz	sc-1647	Mouse monoclonal	1:6000
GSK3	Santa Cruz	sc-7291	Rabbit polyclonal	1:8000
p-IR (Tyr^{1158})	Invitrogen	44-802G	Rabbit polyclonal	1:7000
p-GSK3 α/β ($Tyr^{279}/^{126}$)	UPSTATE	05-413	Rabbit monoclonal	1:3000
Actin	Dr. J.M. Hernandez Cell Biology Department, CINVESTAV, Mexico	----------	Mouse monoclonal	1:10000
**Secondary antibodies**	**Source**	**Catalog no.**	**Clone**	**Dilutions**
Goat IgG-HRP	Jackson ImmunoResearch	305-035-003	Rabbit polyclonal	1:6000
Mouse IgG-HRP	Santa Cruz	sc-2005	Goat polyclonal	1:12000
Rabbit IgG-HRP	Santa Cruz	sc-2004	Goat polyclonal	1:15000

Supplementary Table S2. Activation of the insulin pathway in hepatic cells (Figure 1).

Panel	Proteins	Time (min)					
		0	2	5	10	20	30
A	pIR/actin (C9)	0.731	1.580	1.777	2.040	2.057	2.699
	pIR/actin (H1-6)	0.049	0.493	0.667	1.082	0.920	0.779
B	pIRS/actin (C9)	0.026	0.789	1.021	1.191	1.410	1.093
	pIRS/actin (H1-6)	0.003	0.568	0.959	1.195	0.706	0.695
C	pAkt/actin (C9)	0.008	1.642	1.626	1.550	1.443	0.992
	pAkt/actin (H1-6)	0.029	0.658	1.072	1.493	1.242	1.174
D	pERK 1/2/actin (C9)	0.046	1.366	1.394	0.788	0.679	0.680
	pERK 1/2/actin (H1-6)	0.575	1.880	1.523	0.986	1.199	1.462

Normalization of phosphorylated proteins to actin (housekeeping protein). Values are expressed as arbitrary units of densitometry.

Supplementary Table S3. Effect of RSV on insulin-induced phosphorylation of IR/IRS/Akt and ERK in C9 cells (Figure 2).

Panel	Proteins	Con	Ins	RSV [μM] + Ins [100 nM]			
				12.5	25	50	75
A	pIR/actin (C9)	0.316	1.273	0.901	0.123	0.056	0.115
B	pIRS/actin (C9)	0.007	0.425	1.087	1.594	1.643	0.812
C	pAkt/actin (C9)	0.005	0.982	0.840	0.354	0.215	0.004
D	pERK 1/2/actin (C9)	0.029	0.586	0.957	1.951	2.175	2.294

Normalization of phosphorylated proteins to actin (housekeeping protein). Values are expressed as arbitrary units of densitometry.

Supplementary Table S4. RSV affects time-dependent insulin-induced phosphorylation of IR/Akt (Figure 3).

Panel	Proteins	Con	Ins	RSV [75 μM] + Ins [100 nM]			
				5'	10'	20'	30'
A	pIR/actin (C9)	0.015	1.869	1.616	1.212	0.463	0.465
B	pAkt/actin (C9)	0.005	1.435	1.082	0.791	0.047	0.034
C	pERK1/2/actin (C9)	0.043	0.894	1.359	1.572	1.863	2.205

Normalization of phosphorylated proteins to actin (housekeeping protein). Values are expressed as arbitrary units of densitometry.

Supplementary Table S5. Effect of RSV on insulin-induced phosphorylation of IR/IRS/Akt and ERK in Hepa 1-6 cells (Figure 4).

Panel	Proteins	Con	Ins	RSV [μM] + Ins [100 nM]				
				12.5	25	50	75	100
A	pIR/actin (H1-6)	0.0023	1.2836	0.9460	1.0586	0.7068	0.5209	0.6641
B	pIRS/actin (H1-6)	0.0080	0.9789	1.4136	2.0060	1.8113	1.9344	1.5577
C	pAkt/actin (H1-6)	0.0039	1.1975	1.1442	1.1144	0.2768	0.1997	0.1385
D	pERK 1/2/actin(H1-6)	1.6533	2.1430	1.9529	1.8110	0.6620	0.4334	0.3007

Normalization of phosphorylated proteins to actin (housekeeping protein). Values are expressed as arbitrary units of densitometry.

Supplementary Table S6. RSV negatively regulates the insulin pathway downstream of Akt (Figure 5).

Panel	Proteins	Con	Ins	RSV [μM] + Ins [100 nM]			
				12.5	25	50	75
A,D	pAkt Thr308/actin (C9)	0.651	0.927	0.800	0.275	0.148	0.124
B	pGSK3α/β/actin (C9)	0.548	1.420	0.921	1.070	0.562	0.517
C	pGS Ser641/actin (C9)	0.353	0.347	1.109	1.065	1.084	1.083
D	pAkt Ser473/actin (C9)*	0.005	0.982	0.840	0.354	0.215	0.004

Normalization of phosphorylated proteins to actin (housekeeping protein). Values are expressed as arbitrary units of densitometry. *Normalization values also correspond to Supplementary Table S3-Panel C, since it corresponds to the same experiment.

Supplementary Table S7. Role of PKC in RSV-mediated regulation of insulin signaling (Figure 6).

Panel	Proteins	Con	Ins	RSV [75 μM] + Ins [100 nM]		
				-	BIM	Gö
A	pAkt/actin (C9)	0.109	0.810	0.116	0.745	0.849
C	pERK 1/2/actin (C9)	0.049	0.560	1.143	0.095	0.086

Panel	Proteins	Con	BIM	Ins	RSV [μM] + Ins [100 nM]			
					12.5	100	BIM	Gö
B	pAkt/actin (H1-6)	0.022	0.247	0.998	0.888	0.098	0.599	0.735
D	pERK 1/2/actin(H1-6)	0.314	0.468	0.981	0.574	0.024	0.777	0.987

Normalization of phosphorylated proteins to actin (housekeeping protein). Values are expressed as arbitrary units of densitometry.

Supplementary Table S8. RSV promotes IR-Ser-phosphorylation through PKC activation (Figure 7).

Panel	Proteins	Con	Ins	RSV [75 µM]+Ins [100 nM]			
				**5'**	**10'**	**20'**	**30'**
**Single**	pIR-Ser/actin (C9)	0.007	0.260	0.515	0.630	0.474	0.500

Normalization of phosphorylated proteins to actin (housekeeping protein). Values are expressed as arbitrary units of densitometry.

Supplementary Table S9. Activation of PTP1B by RSV (Figure 8).

Panel	Proteins	Time (min)					
		**0**	**2**	**5**	**10**	**20**	**30**
**A**	pPTP1B/actin (C9)	0.101	0.525	0.879	0.832	0.957	0.968

Panel	Proteins	Con	Ins	RSV [75 µM] + Ins [100 nM]			
				**5'**	**10'**	**20'**	**30'**
**B**	pPTP1B/actin (C9)	0.180	0.288	0.760	0.759	1.083	1.034

Panel	Proteins	Con	Ins	RSV [µM] + Ins [100 nM]			
				**12.5**	**25**	**50**	**75**
**C**	pPTP1B/actin (C9)	0.317	0.664	1.281	1.931	1.972	1.829

Normalization of phosphorylated proteins to actin (housekeeping protein). Values are expressed as arbitrary units of densitometry.
